# Two-stage conversion of syngas and pyrolysis aqueous condensate into L-malate

**DOI:** 10.1186/s13068-024-02532-2

**Published:** 2024-06-21

**Authors:** Alberto Robazza, Flávio C. F. Baleeiro, Sabine Kleinsteuber, Anke Neumann

**Affiliations:** 1 Institute of Process Engineering in Life Sciences 2: Electro Biotechnology, Karlsruhe Institute of Technology - KIT, 76131 Karlsruhe, Germany; 2 Department of Microbial Biotechnology, Helmholtz Centre for Environmental Research – UFZ, 04318 Leipzig, Germany

**Keywords:** Mixed cultures, Energy recovery, Gas fermentation, Carbon capture, Detoxification, Pyrolysis wastewater

## Abstract

Hybrid thermochemical–biological processes have the potential to enhance the carbon and energy recovery from organic waste. This work aimed to assess the carbon and energy recovery potential of multifunctional processes to simultaneously sequestrate syngas and detoxify pyrolysis aqueous condensate (PAC) for short-chain carboxylates production. To evaluate relevant process parameters for mixed culture co-fermentation of syngas and PAC, two identical reactors were run under mesophilic (37 °C) and thermophilic (55 °C) conditions at increasing PAC loading rates. Both the mesophilic and the thermophilic process recovered at least 50% of the energy in syngas and PAC into short-chain carboxylates. During the mesophilic syngas and PAC co-fermentation, methanogenesis was completely inhibited while acetate, ethanol and butyrate were the primary metabolites. Over 90% of the amplicon sequencing variants based on 16S rRNA were assigned to *Clostridium* sensu stricto 12. During the thermophilic process, on the other hand, *Symbiobacteriales*, *Syntrophaceticus*, *Thermoanaerobacterium, Methanothermobacter* and *Methanosarcina* likely played crucial roles in aromatics degradation and methanogenesis, respectively, while *Moorella thermoacetica* and *Methanothermobacter marburgensis* were the predominant carboxydotrophs in the thermophilic process. High biomass concentrations were necessary to maintain stable process operations at high PAC loads. In a second-stage reactor, *Aspergillus oryzae* converted acetate, propionate and butyrate from the first stage into L-malate, confirming the successful detoxification of PAC below inhibitory levels. The highest L-malate yield was 0.26 ± 2.2 mol_L-malate_/mol_carboxylates_ recorded for effluent from the mesophilic process at a PAC load of 4% v/v. The results highlight the potential of multifunctional reactors where anaerobic mixed cultures perform simultaneously diverse process roles, such as carbon fixation, wastewater detoxification and carboxylates intermediate production. The recovered energy in the form of intermediate carboxylates allows for their use as substrates in subsequent fermentative stages.

**Supplementary Information:**

The online version contains supplementary material available at 10.1186/s13068-024-02532-2.

## Background

The fast pyrolysis of lignocellulosic biomass generates bio-char, bio-oil and two by-products: pyrolysis syngas and pyrolysis aqueous condensate (PAC). These by-products contain up to 60% of the carbon of the original biomass [1, 2]. Their composition varies depending on the source material and the pyrolysis process conditions, such as the residence time, pressure, temperature and heating rate [3]. Generally, pyrolysis syngas consists of CO, CO_2_, CH_4_, H_2_, with low concentrations of alkanes and alkenes. Syngas fermentation by acetogenic microorganisms is an attractive technology because it has the potential to reduce carbon emissions from C1-rich exhaust gases while simultaneously producing valuable platform chemicals or biofuels. Axenic processes (*i.e.*, fermentation processes using pure cultures) have been already established at the industrial level, and ongoing research is focused on process optimization and strain development to improve the potential of this technology [4]. On the other hand, PAC contains high concentrations of organic acids, phenolics, aldehydes, ketones, furans, N-heterocyclic and other hazardous compounds. Some PAC components are harmful even at low concentrations, making PAC a challenging substrate for biological conversion [1, 5, 6]. Nonetheless, new technological developments are necessary to improve the overall efficiency of the pyrolysis process, maximizing the recovery of carbon and energy stored in syngas and PAC.

Techno-economical assessments have highlighted the potential of integrating thermochemical and biological processes to improve carbon and energy recovery and to minimize the environmental impact of agricultural wastes [7–11]. In recent studies focusing on the biochemical conversion of some PAC components, researchers combined physiochemical pre-treatments with axenic fermentations to produce a wide range of products [12–16]. Others have utilized the diversity and functional redundancy of the microbial network in the anaerobic digestion process to convert PAC components into biogas. However, methanogenesis was severely inhibited by the toxicity of PAC, leading to the accumulation of short-chain carboxylates in the medium [17, 18]. To improve methane production, recent efforts have successfully employed community enrichment or biochar amendments [5, 6, 17, 19–28]. The taxonomic profiling of the anaerobic communities acclimatized to different PACs highlighted the relevance of syntrophic and acidogenic microorganisms during PAC components degradation [18, 20, 29–37] and their enrichment/bio-augmentation might be a possible strategy to improve PAC degradation and methane production [2].

In an alternative approach to waste methanation, acetate and other carboxylic acids are the primary products of anaerobic fermentation and could subsequently be utilized as feedstock in secondary bioprocesses. Methane is the most favored product with the lowest free energy content per electron ensuring the highest carbon and energy recovery from organic wastes [38, 39]. Thus, methane-arrested anaerobic fermentation for carboxylate production is only achievable by using specific methanogenesis inhibitors [40, 41] or by a specific process design that suppresses the competitiveness of methanogenic pathways [42]. For instance, researchers have been using CO at high partial pressures [43–45] to inhibit methanogens or low pH to increase the concentrations of undissociated carboxylic acids [46, 47]. Some other studies successfully attempted to exploit the toxicity of PAC to inhibit or mitigate methanogenic microorganisms, allowing for carbon and energy recovery from PAC and syngas into carboxylates [18, 48]. Acetate and other carboxylic acids can be used as intermediate substrates in two-stage (anaerobic to aerobic) biological processes to produce high value chemicals from syngas and wastewaters. Combining two bioprocesses into sequential fermentations with carboxylates as intermediates is considered a promising approach for the production of high-energy-density and high-value chemicals from waste streams [49–55]. However, when dealing with biological processes to treat toxic wastewater, the success of the second fermentation stage depends on the detoxification achieved in the first stage. Besides improving toxicant removal rates in the first stage, the selection of appropriate microorganisms for the second stage might affect process performance and carboxylate conversion rates [48]. Fungi have been reported to be the microorganisms most tolerant to the oil fraction of the condensates from pyrolysis [56], and the tolerance of *Aspergillus oryzae* to PAC and some selected PAC components has been characterized before [48, 57, 58]. *A. oryzae* is known for its metabolic versatility to grow on sugars and carboxylic acids present in various waste streams for the production of single cell proteins [59–62] or L-malate [63–65], among other chemicals. L-Malate has a wide array of applications, ranging from taste-enhancer in the food industry to biopolymer production [66]. In 2004, L-malate was regarded as one of the 12 most important biomass-derived biochemical [67]. In 2020, the annual global L-malate production was estimated to be around 80 000 to 100 000 tons, while the market demands up to 200 000 tons per year, a value expected to increase in the following years [66]. L-Malate production from non-food feedstock could be an economical and efficient way to meet market needs [68]. L-Malate production from PAC was viable only after an extensive physiochemical detoxification process [57, 69]. Biological detoxification via anaerobic mixed cultures, on the other hand, has been proven to be a valid alternative to reduce PAC components’ toxicity below inhibitory levels while producing carboxylic acids [48].

However, very limited knowledge is available about the continuous co-fermentation of syngas and PAC by anaerobic mixed cultures in stirred tank reactors (STRs) for short-chain carboxylates production. In this work, a two-stage sequential fermentation process was tested, where the products of the anaerobic fermentation of syngas and PAC were valorized to produce L-malate with *A. oryzae*. Two mesophilic (37 °C) and thermophilic (55 °C) enrichment processes were run in identical semi-continuous STRs at slightly acidic pH and increasing PAC loading rates to evaluate the effects of temperature and PAC on the process performances and on the microbial community composition. In the second stage, the effluent from the first-stage fermentation was inoculated with *A. oryzae* to grow on the acetate, propionate and butyrate to produce L-malate.

## Materials and methods

### Inocula and PAC

The anaerobic sludge was collected at a biogas reactor treating cow manure and handled as described in a previous work [48]. The total solids, total fixed solids and total volatile solids of the anaerobic sludge were 79.6 ± 0.5 g/L, 26.1 ± 1.1 g/L, and 53.5 ± 0.7 g/L, respectively, as determined following Method 1684 [70]. *A. oryzae* DSM 1863 was provided by the DSMZ strain collection (Deutsche Sammlung von Mikroorganismen und Zellkulturen GmbH, Braunschweig, Germany). The lignocellulose PAC was generated during the fast pyrolysis of miscanthus at the BioLiq plant (Karlsruhe Institute of Technology, Karlsruhe, Germany). The GC–MS analysis of the PAC performed by the Thünen Institute of Wood Research (Hamburg, Germany) is available in Additional file 1 (Table S1).

### Mixed culture enrichments

The enrichments were performed in two identical 2.5-L semi-continuous stirred tank reactors (Minifors, Infors HT, Bottmingen, Switzerland) with a working volume of 1.5 L. The bioreactor design was already optimized for gas fermentation [50]. The cultivations were carried out at 37 °C or 55 °C, 500 rpm, pH 5.5, and atmospheric pressure. The pH was monitored online with an EasyFerm Plus PHI K8 225 (Hamilton Bonaduz AG, Bonaduz, Switzerland) and controlled with 4 M NaOH or 4 M H_3_PO_4_ solutions. Changes in the oxidation–reduction potential of the broth were measured with the ORP sensor Polilyte Plus ORP Arc 225 (Hamilton Bonaduz AG, Bonaduz, Switzerland) and used as an indicator of metabolic activity and to control for air contamination. A synthetic syngas consisting of about 3 kPa H_2_, 25 kPa CO_2_, and 20 kPa CO in N_2_ was fed at a gassing rate of 18 mL/min (0.012 vvm). The gases were controlled individually via mass flow controllers (red-y smart series, Vögtlin, Muttenz, Switzerland), and injected via a micro-sparger into the vessel. The fermentation broth and the feed were composed of a modified basal anaerobic (BA) medium and PAC. The composition of the modified BA medium is available in Additional file 1. The BA medium for the feed bottles was poured into 2-L glass bottles (Schott AG, Mainz, Germany), autoclaved, flushed and pressurized with N_2_ up to 0.5 bar to make it anoxic and prevent oxygen leaks. One milliliter per liter of a 100 g/L cysteine solution was added into the feed bottles as a reducing agent and sulfur source. After autoclaving, the feed bottles and the bioreactors were connected by platinum-cured silicone tubing of 1.6 mm wall thickness (Watson Marlow, Bergenfield, New Jersey, USA). The PAC was poured into a 2-L glass bottle, made anoxic, and stored at 4 °C. During continuous operations, the BA medium and PAC were injected at the same time of the day to achieve a total average feed rate of 75 mL/d, resulting in a hydraulic retention time (HRT) of 20 days. Depending on the PAC loading, the required volume of PAC was withdrawn from the bottle with a syringe and then injected into the bioreactor via a silicon septum on the head plate of the bioreactor. The PAC loadings in the feed were 1% (2.53 gCOD/L), 2% (5.06 gCOD/L), 3% (7.59 gCOD/L), 4% (10.13 gCOD/L), 5% (12.66 gCOD/L), 6% (15.19 gCOD/L). Additional information about the feed composition and load (of syngas and PAC) are available in Additional file 1 (Table S2). Except for PAC loadings of 1% and 2%, each loading of PAC was maintained constant for a period corresponding to at least 40 days (*i.e.*, twice the HRT).

Before the first inoculation, 15 mL PAC (1% v/v) was injected into the bioreactors and the pH was adjusted to 5.5. Both bioreactors were inoculated with 400 mL inoculum (27% v/v). Only after the first inoculation, due to the buffering capacity of the inoculum, the pH rose up to 6.7 but lowered naturally at 0.1 per day to the desired pH of 5.5.

If both the CO partial pressure at the gas outlet and the ORP value were increasing close to 20 kPa and above -100 mV, respectively, then the bioreactor was re-inoculated with the original inoculum to reach about 12 g/L of total suspended solids (TSS). If any of the bioreactors required several inoculation events, the TSS was controlled in the range of 8–12 g/L by weekly re-inoculations with on average of 75 mL of inoculum. The TSS and volatile suspended solids (VSS) were determined as explained in Additional file 1.

### Bioreactor sampling and analytical methods

Five milliliters of the liquid phase of the bioreactors were sampled daily, collected in 15 mL pre-weighed Falcon tubes, and centrifuged at 14,000 × *g* for 1 h.

The supernatant was collected, filtered, and stored at −20 °C for later analyses. The pellet was dried for 24 h at 80 °C and used to determine the total suspended solids. The concentrations of formate, acetate, propionate, *n-*butyrate (from here onwards defined as short-chain carboxylates, SCCs), L-malate and ethanol together with the concentrations of few selected PAC components (furfural, phenol, guaiacol, and *o-*, *m-*, *p*-cresol) were determined by a high-performance liquid chromatography (HPLC) device run as described previously [48].

The online determination of the fractions of CO, H_2_, CO_2_, N_2_, O_2_, and CH_4_ in the gas phase of the bioreactors was performed via gas chromatography (GC) using a GC-2010 Plus AT (Shimadzu, Japan) with a thermal conductivity detector equipped with a ShinCarbon ST 80/100 column (2 m × 0.53 mm ID, Restek, Germany) and an Rtx-1 capillary column (1 µm, 30 m × 0.25 mm ID, Restek, Germany) with helium as carrier gas. Assuming N_2_ to be biologically inert and the inflowing gas composition constant throughout the whole fermentation period, it was possible to compute the molar consumption and production of gaseous substrates and products via the ideal gas law as explained in a previous work [50]. Electron mole (e-mol) recovery was used to calculate the chemical fluxes in the process. The e-mol recovery is the ratio between the daily cumulated e-mol production of H_2_, CH_4_, formate, acetate, propionate and butyrate and the daily e-mol fed into the bioreactors as syngas and PAC. Further details of the calculations are described in Additional file 1. Table S3 in Additional file 1 lists the metabolites and their conversion factors for the e-mol recoveries. All the other calculations are available in Additional file 1.

### Microbial community analysis and statistical evaluation

Every 20 days or before and after any inoculation event, technical duplicates of 2 mL of fermentation broth were sampled and centrifuged for 30 min at 17,000 × *g*. After discarding the supernatant, the pellet was re-suspended in 1 mL phosphate-buffered saline solution (pH 7.4). The pellets from both samples were combined and centrifuged for another 30 min at 17,000 × g. The pellets were stored at −20 °C. Details on the procedures for DNA extraction, sample purification, PCR, and description of the amplification primers are described previously [41]. Amplicon sequencing of the 16S rRNA (region V3–V4) and mcrA genes was done using the Illumina MiSeq platform. Library preparation for the visualization of the microbial community and elaboration of Spearman correlations was performed as described in another work [71]. The raw sequence data without adapters used in this study have been deposited in the European Nucleotide Archive (ENA) under the study accession number PRJEB72504 (http://www.ebi.ac.uk/ena/data/view/PRJEB72504).

### *Aspergillus oryzae* batch fermentations

At every increase in PAC loading, 50 mL broth were withdrawn from the bioreactors and centrifuged for 1 h at 14,000 × g. The pH of the supernatant was corrected to 6.5 with 4 M NaOH solution. Nine milliliters of the supernatant together with 1 mL BA medium were poured into 100-mL baffled Erlenmeyer flasks and inoculated with 0.1 mL of *A. oryzae* conidia (spore concentration of 3 × 10^7^ spores/mL). The shaking flasks were incubated at 30 °C and 100 rpm. The aqueous phase (0.2 mL) was sampled daily and controlled for pH; the concentrations of SCCs and L-malate concentrations were determined with HPLC. All fermentations with *A. oryzae* were performed in triplicates.

## Results

Initially, syngas and PAC were co-fermented by two mixed microbial cultures at 37 °C and 55 °C in semi-continuous STRs to test the carbon and energy recovery potential from the two pyrolysis process streams. Some samples of the supernatant of the fermentation broth from the bioreactors were inoculated with *A. oryzae* to produce L-malate from the carboxylates.

### Mesophilic co-fermentation of syngas and PAC

**Fig. 1:**
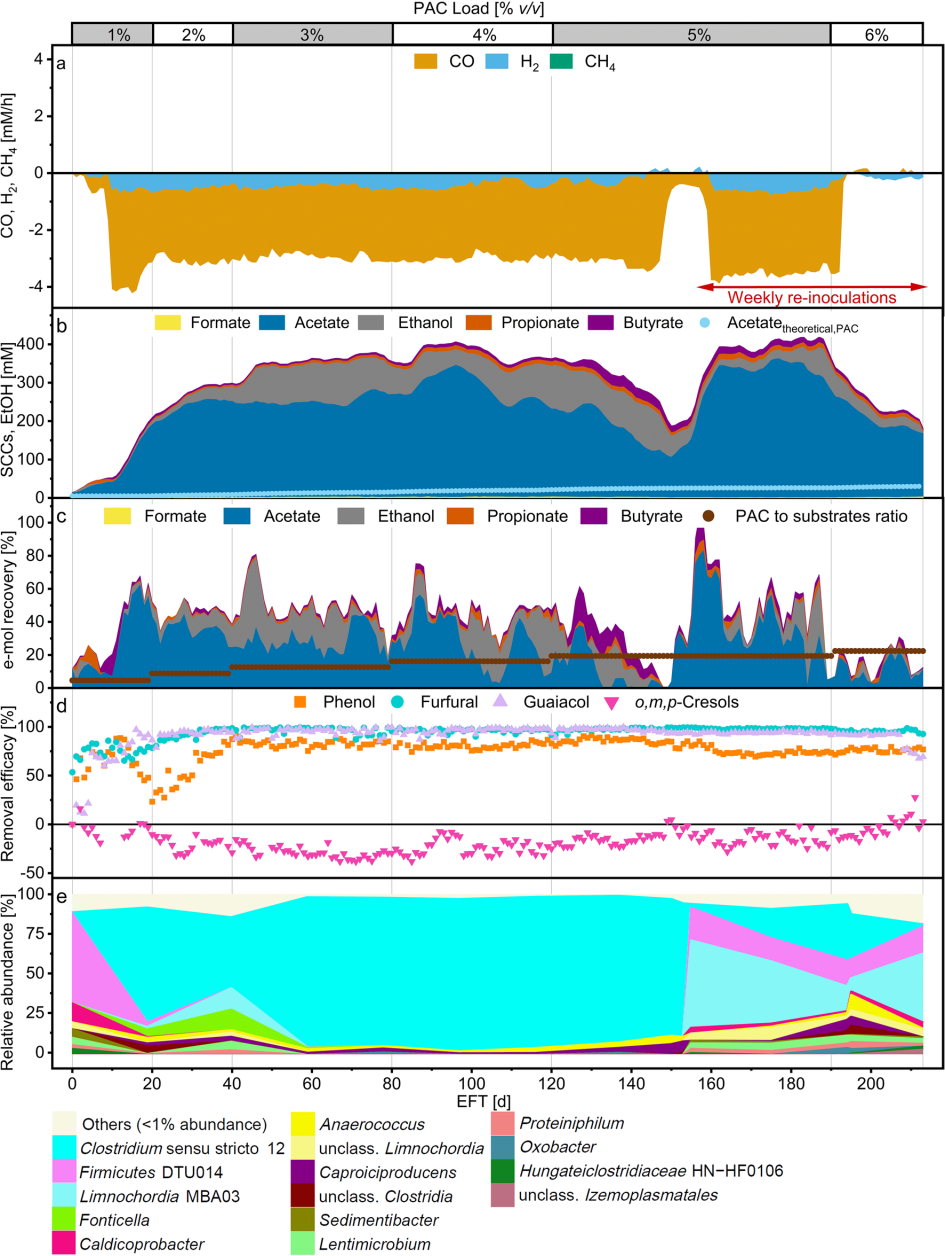
Fermentation profile of the mesophilic process. Top x-axis marks the PAC loading, bottom x-axis shows the elapsed fermentation time (EFT). The red bar indicates the period of weekly re-inoculations. **a** Consumption and production rates of gaseous compounds. Negative values indicate consumption. **b** Formate, acetate, propionate and butyrate (SCCs) and ethanol concentrations in the fermentation broth. Acetate_theoretical, PAC_ is the theoretical acetate concentration from PAC. **c** Daily e-mol recovery into products from syngas and PAC fed; daily ratio of e-mol of PAC in the feed to total e-mol of syngas and PAC fed (brown). **d** Removal efficacies of selected PAC components. Negative efficacy values indicate production. **e** Relative abundance of the enriched microbial genera (based on 16S rRNA amplicon sequencing variants)

Overall, the mesophilic reactor showed stable performance throughout most of the fermentation period. Figure 1 reports the combined graphs of the relevant parameters for the mesophilic reactor treating syngas and PAC at increasing PAC loadings. CO consumption started 10 days after the inoculation of the reactor. After peaking at 4.2 mM/h on day 15, CO consumption rates were about 3 mM/h until day 145 (Fig. 1a). Concomitantly, exogenous H_2_ consumption remained relatively stable at about 0.45 mM/h. No methane production was detected. The redox potential of the medium ranged between −360 and −380 mV (Additional file 1, Figure S1a). The partial pressures of CO and H_2_, together with redox potential and the pH of the medium are shown in Additional file 1 (Figure S1b). From day 20 to day 145, CO partial pressure averaged to about 10 kPa while the H_2_ partial pressure did not exceed 3 kPa. Acetate and ethanol were the primary metabolites detected in the fermentation broth (Fig. 1b), with selectivities ranging between 44 and 92% and 2–42%, respectively. From day 150 on, the acetate concentration increased to about 350 mM within less than 10 days.

Small amounts of butyrate (up to 30 mM) were detected between days 120 and 150. Small amounts of propionate were also found. The concentrations of undissociated acids are shown in Additional file 1 (Figure S1c). Acetic acid concentration never exceeded 60 mM while butyric acid concentration was always below 10 mM. Phenol, furfural and guaiacol removal reached efficacies higher than 80% within the first 40 days of fermentation and remained stable until the end of the fermentation (Fig. 1d). The cumulative removal of *o-, m-, p*-cresols was negative. *m*- and *p*-cresols were produced, whereas *o*-cresol was the only cresol that showed consistent removal efficacy (Additional file 1, Figure S1c). Between day 10 and day 120, the mesophilic enrichment recovered on average about 50% into SCCs and ethanol of the total e-mol of syngas and PAC fed daily (Fig. 1c). For the mesophilic process, the e-mol recovery accounts for SCCs only as products (H_2_ is a substrate), not including longer-chain carboxylates (with high electron equivalents) and biomass production. From about day 120, the e-mol recovery decreased to values close to 0 on day 145. Then, the CO consumption rates decreased sharply concomitant with an increase of the redox potential to about −150 mV. This result suggests that the carboxydotrophic portion of the reactor microbiota (*i.e.*, microbes contributing to CO consumption) underwent severe stress. Decreasing CO consumption rates were not accompanied by changes in the PAC components removal, which remained somewhat constant. To recover syngas metabolism, the fermenter was re-inoculated with about 350 mL of inoculum (sufficient to reach at least 12 gTSS/L) to increase biomass concentration and microbial diversity within the bioreactor. About 4 days after the first re-inoculation event, CO consumption rates recovered. From day 150, the amount of VSS was maintained within 1 to 5.6 g/L by regular re-inoculations (Additional file 1, Figure S1d). After 195 days, CO and H_2_ conversion rates dropped to zero and never recovered, suggesting that PAC loads of 15.19 gCOD/L/d are too high to maintain carboxydotrophic activity.

From day 20 to day 150, the reactor microbiota (Fig. 1e) was dominated by five amplicon sequencing variants (ASVs) (ASV 002, 005, 010, 012 and 023) belonging to *Clostridium* sensu stricto 12 (over 90% abundance), a genus that comprises acetogenic microorganisms such as *Cl. ljungdahlii* and *Cl. autoethanogenum*. Other microorganisms enriched during this period were belonging to the genera *Anaerococcus* (ASV 018) and *Caproiciproducens* (ASV 028). From about day 100, the cumulative relative abundance of *Anaerococcus* and *Caproiciproducens* spp. increased up to about 10% at the expense of *Clostridium* sensu stricto 12. At the same time, acetate and ethanol concentrations decreased while butyrate concentration increased. Increasing abundance of *Anaerococcus* and *Caproiciproducens* coincided with the decrease in e-mol recovery. From day 150, the abundance of *Firmicutes* DTU014, *Limochordia* MBA03*, Caldicoprobacter* (ASV 018) and two unclassified *Limnochordia* species (ASV 016 and 019) increased as results of the weekly re-inoculations. Similarly, *Clostridium* sensu stricto 12 and *Caproiciproducens* abundance recovered up to about 40% and about 5%, respectively.

**Fig. 2:**
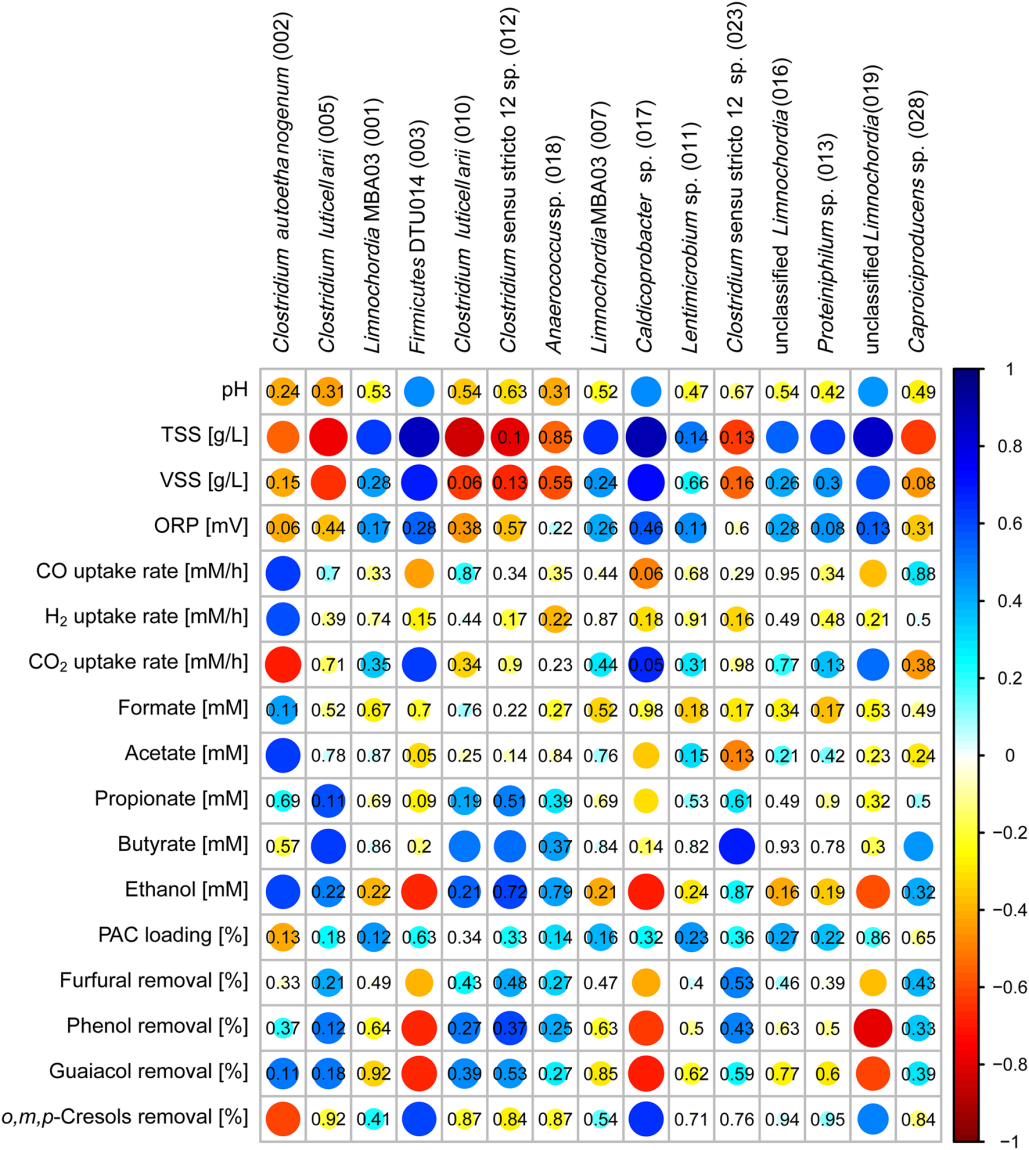
Spearman’s rank correlations between relative abundance of dominant amplicon sequencing variants (ASVs) and process parameters for the mesophilic semi-continuous STR enrichment. The strength of the correlation is represented by the size of the circle and intensity of the color. Blue circles indicate positive correlations. Red circles indicate negative correlations. *p* values are shown for non-significant correlations (*p* > 0.05)

Significant correlations (*p* < 0.05) (Fig. 2) suggest that ASV 002, a close relative of *Clostridium autoethanogenum* (and consequently of its relatives: *Cl. ragsdalei*, *Cl. coskatii*, and *Cl. ljungdahlii*), represents the primary carboxydotroph converting syngas into acetate and ethanol. Two members of *Clostridium luticellarii* (ASV 005 and 010) and other ASVs assigned to the genera *Caproiciproducens* (ASV 028) and *Clostridium* sensu stricto 12 (ASV 012 and 023) likely contributed to butyrate production. Abundance of *Clostridium autoethanogenum* ASV 002 was negatively correlated to cresol removal, whereas members of *Firmicutes* DTU014 (ASV 003)*, Caldicoprobacter* (ASV 017) and an unclassified *Limnochordia* sp. (ASV 019) showed significant correlations to cresol removal (*p* < 0.05).

### Thermophilic co-fermentation of syngas and PAC

**Fig. 3:**
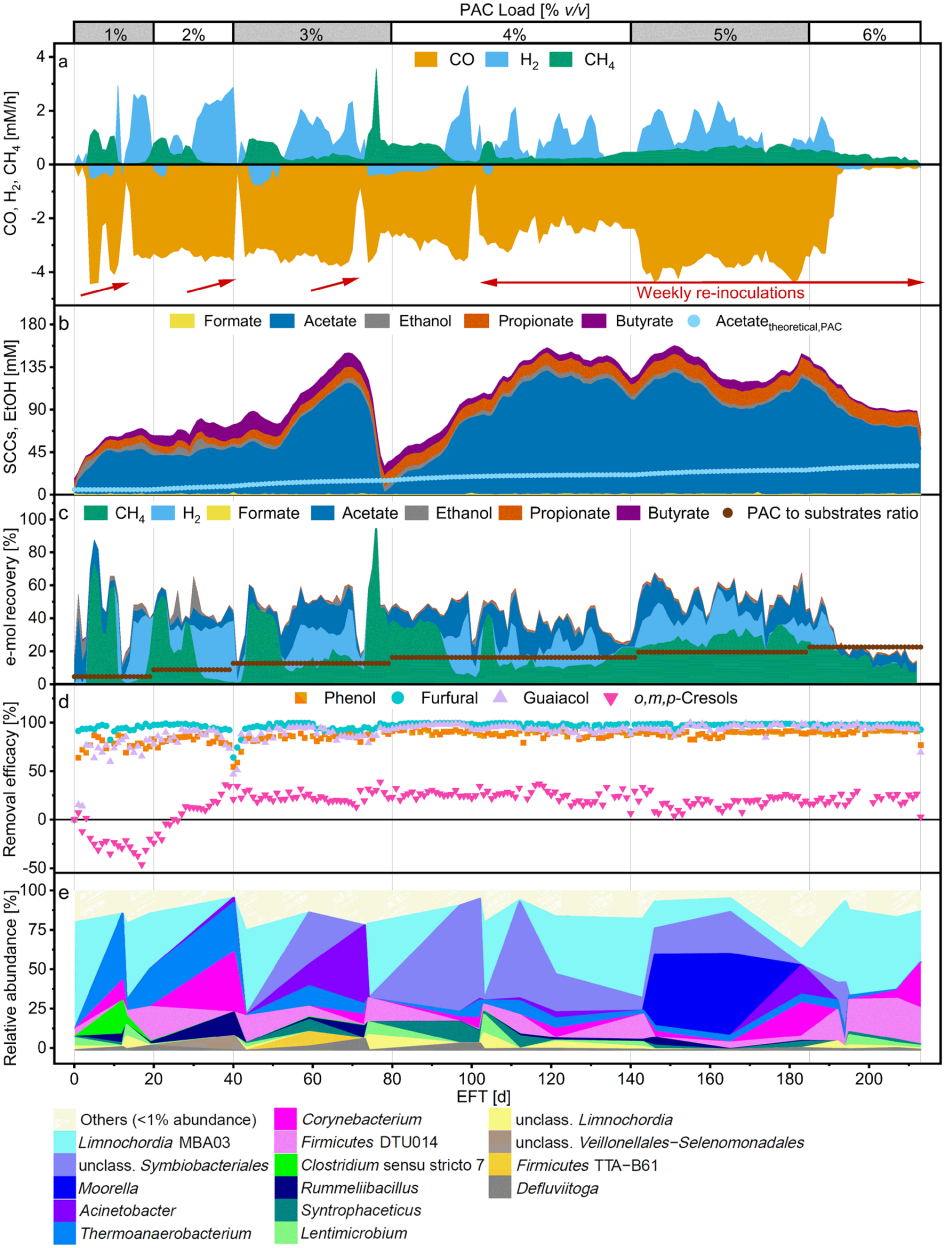
Fermentation profile of the thermophilic process. Top x-axis marks the PAC loading, bottom x-axis shows the elapsed fermentation time (EFT). Red arrows point to re-inoculation events, the red bar indicate the period of weekly re-inoculations. **a** Consumption and production rates of gaseous compounds. Negative values indicate consumption. **b** Formate, acetate, propionate and butyrate (SCCs) and ethanol concentrations in the fermentation broth. Acetate_theoretical,PAC_ is the theoretical acetate concentration from PAC. **c** Daily e-mol recovery into products from syngas and PAC fed; daily ratio of e-mol of PAC in the feed to total e-mol of syngas and PAC fed (brown). **d** Removal efficacies of selected PAC components. Negative efficacy values indicate production. **e** Relative abundance of the enriched microbial genera (based on 16S rRNA amplicon sequencing variants)

During the thermophilic co-fermentation, CO consumption was detected within the first 5 days after inoculation. The conversion of syngas followed the stoichiometry of the water-gas shift reaction and of hydrogenotrophic methanogenesis. CO was primarily converted to CO_2_, H_2_ and CH_4_ (Fig. 3a). The carbon equivalents in CO_2_ and CH_4_ accounted on average for 84% of the total carbon equivalents from syngas. Similarly, the electron equivalents in H_2_ and CH_4_ accounted for about 86% of the total electron equivalents from syngas. CO was metabolized at an average rate of 3.1 ± 0.5 mM/h until day 140. Between days 145 and 186, the CO uptake rates increased by 20% to 3.8 ± 0.3 mM/h. H_2_ conversion rates alternated between production and consumption depending on the extent of inhibition of methanogenesis. The CO partial pressures mostly fluctuated around 10 kPa to decrease to about 5 kPa between days 145 and 186. H_2_ partial pressures ranged from 0.4 to 11.9 kPa (Additional file 1, Figure S3b). The redox potential oscillated between −480 and −350 mV (Additional file 1, Supp. Figure 3a). Acetate was the primary SCC produced (with selectivity on average higher than 80% during the whole fermentation period) followed by small concentrations of butyrate, propionate and ethanol (all never exceeding 20 mM throughout the fermentation period). For the first 40 days, acetate concentration remained constant at about 50 mM but later increased up to 118 mM after 70 days. Profiles of the concentrations of the undissociated acids are available in Additional file 1 (Figure S2c). Around day 73, the pH was temporarily increased from 5.5 to 6.7 to test the effect on methanogenesis (Additional file 1, Figure S2a). CH_4_ production rates spiked up to 3.6 mM/h for few days until the acetate was completely consumed and the pH was adjusted back to 5.5. From day 77 onwards, acetate concentration steadily increased up to about 130 mM on day 119. Afterwards, it oscillated between 125.1 mM and 68.2 mM until the end of the fermentation period. Phenol, furfural and guaiacol were removed with high efficacies (Fig. 3d). The cumulative removal of the cresols, after being produced during the first 26 days of fermentation, increased to on average 22.5% for the rest of the fermentation period. The removal efficacy of each cresol isomer is reported in Additional file 1 (Figure S2d). The e-mol recovery reached 100% during the first ten fermentation days but later decreased to about 50%, regardless of the increasing e-mol loading from PAC. From day 145 on, the e-mol recovery increased due to the higher CO uptake rates (Fig. 1c). Methane was the primary e-mol acceptor for the e-mol from syngas and PAC fed into the system.

After the daily feeding events on days 43, 72 and 101 (corresponding to PAC loadings of 3% and 4% v/v), the CO consumption exhibited a sharp decline. CO uptake rates decreased below 0.5 mM/h, within few hours from feeding (zoomed-in profiles of CO, H_2_ and CH_4_ production rates around days 43, 72, 101 are available in Additional file 1, Figure S4). Simultaneously, the redox potential increased to values higher than −100 mV while the VSS were estimated to have fallen below 1 g/L (Additional file 1, Figure S2e). No air contamination was detected. Similarly, an erroneous addition of about 6 mL of PAC on day 10 caused the CO consumption rate to fall. From about day 40, the removal of phenol, furfural and guaiacol decreased for a few days from 90% to about 54%, 64% and 47%, respectively. Decreasing removal of PAC components was not detected again. Decreasing CH_4_ production rates and increasing H_2_ partial pressures forwent the decrease of CO uptake rates. On the days following the decrease of CO consumption rate, the bioreactor was inoculated (indicated by red arrows in Fig. 3a), resulting in a quick recovery of CO consumption rate. Given the success of the re-inoculation, from day 101 onwards, the fermenter was re-inoculated weekly, to maintain high biomass concentrations (the VSS ranged between 6.5 and 1.5 g/L averaging at 3.3 ± 1.0 g/L). The weekly re-inoculation strategy stabilized the reactor performance as no major disturbances of syngas conversion were observed for the following 90 days.

The microbial community analysis showed that *Limnochordia* MBA03, *Firmicutes* DTU014, a *Lentimicrobium* sp. and an unclassified *Limnochordia* species abounded after each inoculation but were progressively washed out after 43, 72 and 101 days (Fig. 3e). On the contrary, bacteria identified as members of *Symbiobacteriales*, *Acinetobacter*, *Thermoanaerobacterium*, *Rummeliibacillus*, *Corynebacterium*, *Syntrophaceticus* and unclassified *Veillonellales–Selenmonadales* were enriched during stable operations. Amplicon sequencing of *mcrA* genes indicated that thermophilic conditions favored the enrichment of *Methanothermobacter. Methanosarcina* spp. and *Methanoculleus* were methanogens abundant in the inoculum but did not perform well in the reactor (Additional file 1, Figure S2f). Weekly re-inoculation of the bioreactor helped also maintain a highly diverse microbiome. From day 101 onwards, the most abundant taxa were *Limnochordia* MBA03, *Symbiobacteriales*, *Acinetobacter*, *Thermoanaerobacterium*, *Corynebacterium* and *Firmicutes* DTU014. Between days 145 and 186, a close relative to *Moorella thermoacetica* was enriched up to 50% abundance, coinciding with higher CO uptake rates. *Methanothermobacter* was consistently enriched also during the re-inoculation phase but its abundance progressively lowered from about 75% after 115 days to about 25% after 213 days in favor of *Methanosarcina* (Additional file 1, Figure S2).

**Fig. 4:**
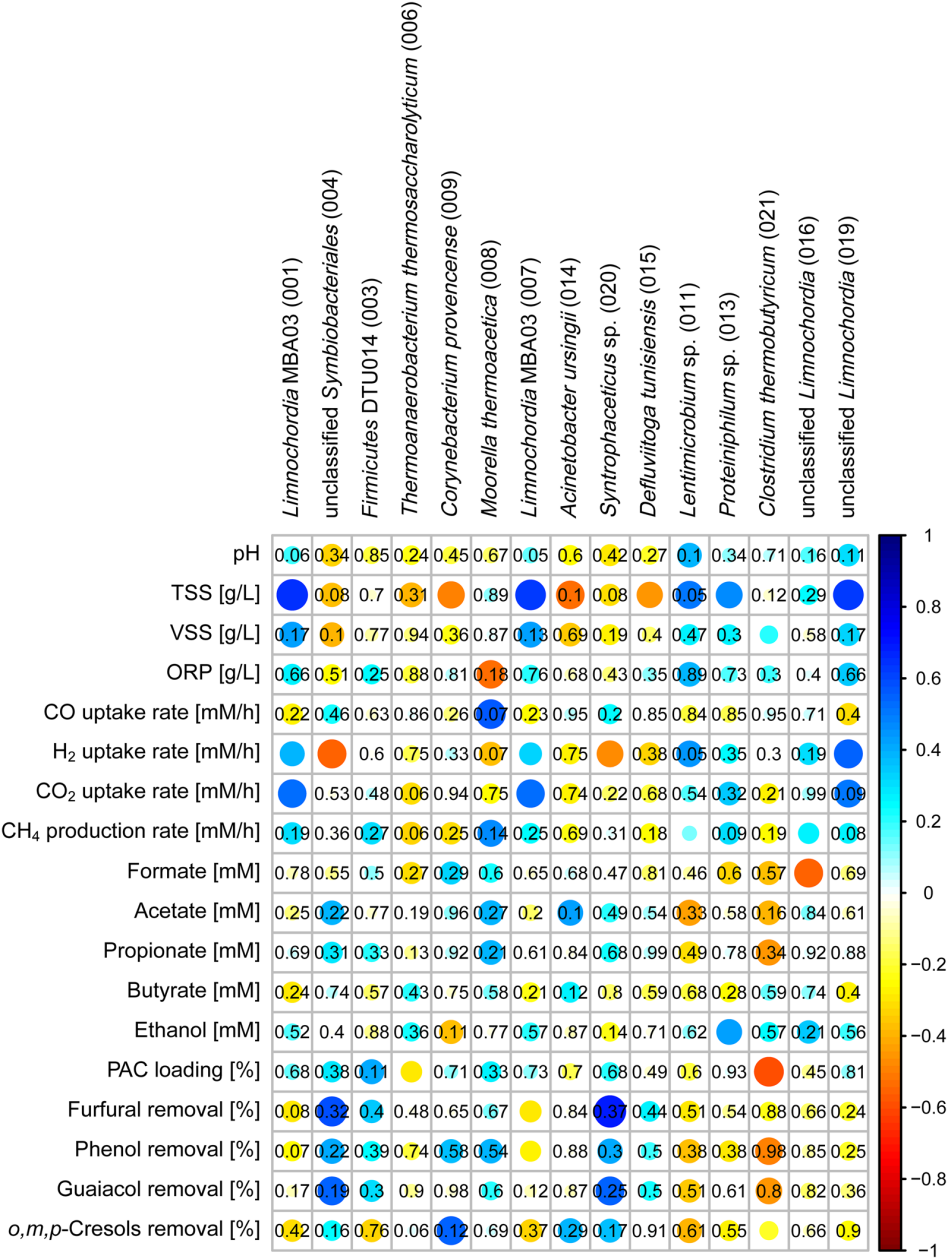
Spearman’s rank correlations between relative abundance of amplicon sequencing variants (ASVs) and process parameters for the thermophilic semi-continuous STR enrichment. The strength of the correlation is represented by the size of the circle and intensity of the color. Blue circles indicate positive correlations. Red circles indicate negative correlations. p values are shown for non-significant correlations (*p* > 0.05)

*Moorella thermoacetica* ASV 008 was the only ASV that showed a strong correlation to CO uptake (albeit with high p-value of 0.07). *Symbiobacteriales* ASV 004 and *Syntrophaceticus* ASV 020 showed significant correlations to hydrogen production (Fig. 4). Two *Methanothermobacter* species (ASVs 001 and 002) showed significant correlation to H_2_ production (*p* < 0.05) while *Methanosarcina thermophila* ASV 003 showed significant correlation to H_2_ consumption (*p* < 0.05) (Additional file 1, Figure S3).

### L-Malate production from SCCs with *A. oryzae*

**Fig. 5:**
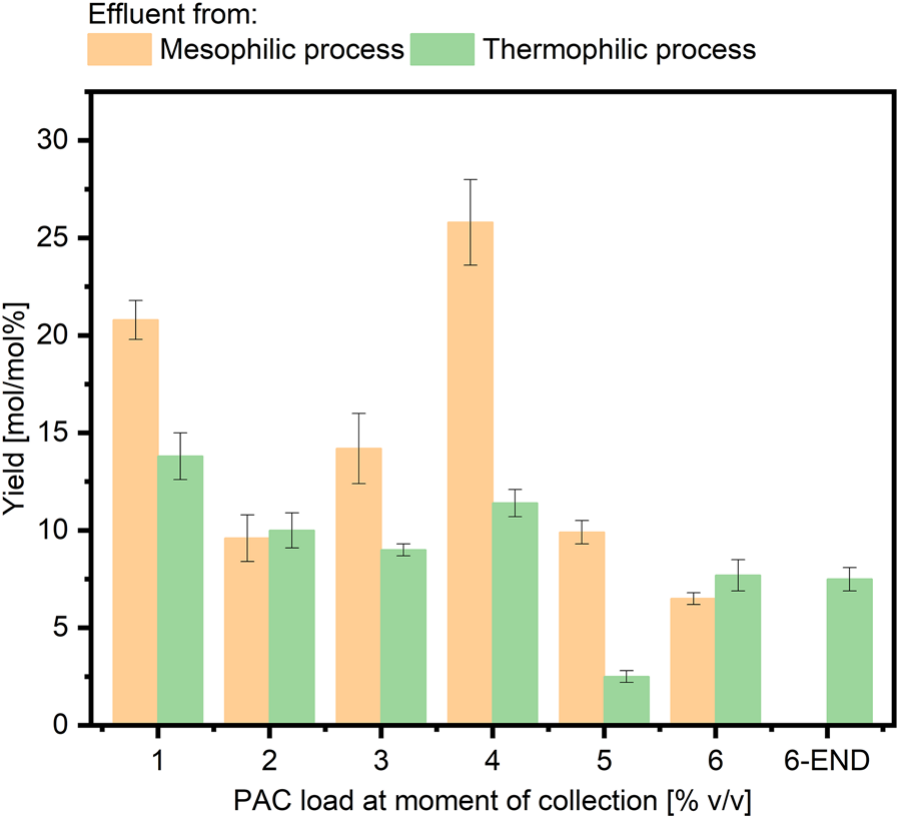
L-Malate yields calculated for the highest L-malate concentrations per SCCs consumed. Bars represent mean values with standard deviations (*n* = 3)

To assess the overall detoxification process of PAC and explore the potential for carboxylate valorization, the effluent originating from both mesophilic and thermophilic fermentations was used as fermentation medium in subsequent aerobic fermentations. Specifically, 50 mL of reactor broth were collected prior to each increment in PAC loading and underwent centrifugation as described in Materials and methods. Subsequently, the resulting supernatant was inoculated with *A. oryzae* conidia. The *A. oryzae* fermentations were categorized based on the origin of the reactor effluent (whether from the mesophilic or thermophilic process) and the specific PAC loading within the reactor at the time of sampling (Fig. 5).

Growth of *A. oryzae* was observed in all batch fermentations but one. *A. oryzae* effectively consumed all the SCCs generated during mesophilic and thermophilic syngas and PAC co-fermentations. An exception was detected with the final sample collected from the mesophilic fermenter where no *A. oryzae* growth nor L-malate production were recorded (Additional file 1, Figure S5). The highest L-malate titer observed was 33.0 ± 0.8 mM, produced by *A. oryzae* from the acetate, propionate and butyrate present in the effluent collected from the mesophilic bioreactor after 80 days (Additional file 1, Table S4 and Figure S6). In contrast, the thermophilic reactor produced a maximum of 13.9 ± 1.7 mM of L-malate from sample collected at 120 days, with a 4% PAC loading (Additional file 1, Table S5 and Figure S7). The highest L-malate yield of all *A. oryzae* fermentations, amounting to 25.8 ± 2.2 mol/mol%, was achieved with the medium collected from the mesophilic bioreactor at 116 days. Overall, the L-malate yields exhibited a decreasing trend as the PAC loading increased in the medium from both reactors.

## Discussion

### Reactor microbiomes and performances of the mesophilic and the thermophilic process

The high abundance of *Clostridium* sensu stricto 12 in the mesophilic process suggests the central role they played during the mesophilic syngas and PAC co-fermentation. The dominant ASV 002 was assigned to *Cl. autoethanogenum,* a well-studied carboxydotrophic acetogen known for its application in companies specializing in syngas fermentation [72]. This specie is known to consume CO and H_2_/CO_2_, yielding acetate and ethanol. The enrichment *Cl. autoethanogenum* was possibly influenced by the reactor design and feed composition. Syngas accounted for over 90% of the total e-mol for the first 40 days of the fermentation.

Although no significant correlation was identified between *Clostridium* sensu stricto 12 and aromatics removal in this work, prior research indicated that *Clostridium* sensu stricto participated in the anaerobic digestion of aromatics-rich wastewaters [73–76]. Examples include also the enrichment of *Clostridium* sensu stricto (up to 17.5%) during the degradation of tars from rice husk gasification, where biochar facilitated syntrophic relations with *Methanosaeta* [26]. Another study documented the enrichment of *Clostridium* sensu stricto 1 and 12 up to 5% abundance during the co-fermentation of syngas and PAC in a packed biochar reactor [18]. Similarly, during the anaerobic digestion of phenol-rich coal gasification wastewater with addition of graphene, about 10% of the rector microbiota was composed of *Clostridium* sensu stricto 5 and *Clostridium* sensu stricto 1 [77].

Here, *Clostridium* sensu stricto 12 may have also been involved in the production of cresol. Even though, some studies reported anaerobic cresol removal, not all cresols exhibit similar removal efficiency [17, 48]. Some other studies described cresol production during the anaerobic digestion of corn straw and within the human intestinal tract [78–80]. A screening of 153 human intestinal bacterial species grown on tyrosine showed that 36 species were able to produce phenol while 55 produced *p-*cresol. Four strains belonging to *Clostridium* sensu stricto 11 and 14a produced 100 mM of *p-*cresol while one strain of *Anaerococcus* produced up to 100 mM *p-*cresol [79]. Although *Firmicutes* DTU014 and *Limnochordia* MBA03 were correlated with cresol removal in this study, there is no evidence supporting it. Their persistence in the system was not stable, as they were gradually washed out of the reactor. *Firmicutes* DTU014, *Limnochordia* MBA03 are slow-growing syntrophic electroactive bacteria commonly found in industrial anaerobic digesters [81, 82]. They have been observed during the thermophilic anaerobic digestion of phenyl acids [83] and during the anaerobic digestion of the aqueous phase of hydrothermal liquefaction [32], but their function still remains unclear.

Other clostridial ASVs affiliated to *Cl. luticellarii*, *Caproiciproducens* and *Clostridium* sensu stricto 12 may have contributed to butyrate production. *Cl. luticellarii* is an acetogenic bacterium that can also produce *n-*butyrate and *iso-*butyrate. Mildly acidic pH (5.5) and 50 mM acetate stimulated *n-*butyrate and *iso-*butyrate production to a selectivity of about 42% [84]. *Cl. luticellarii* was also considered the main candidate for methanol and propionate conversion into valerate in an anaerobic chain elongation open-culture reactor [85]. Similarly, *Caproiciproducens*, a genus commonly found in chain-elongating microbial communities, produces butyrate and caproate [86–88]. The production of longer-chain carboxylic acids, such as valerate and caproate, has been previously documented in the co-fermentation of syngas and PAC at 30 °C [18]. *Anaerococcus*, enriched concurrently with *Caproiciproducens*, can ferment a wide range of carbohydrates, peptone or amino acids to produce carboxylic acids [89] and it has been previously correlated with *iso*-butyrate production in syngas reactors [90]. Only few works have reported the enrichment of *Anaerococcus* during the anaerobic digestion of food waste and swine manure and their function within anaerobic mixed cultures is not clear [78, 91–93]. The conversion of SCCs and electron donors into non-monitored longer-chain carboxylates, may have contributed to the reduction in e-mol recoveries. Alternatively, or possibly in conjunction, the diminishing e-mol recovery may be linked to a concurrent decrease in the degradation rates of PAC components. A decrease in degradation could lead to lower SCCs production rates and increasing toxicant concentrations, which could ultimately cause also a cessation of CO uptake.

The elevated concentrations of undissociated acids, reaching about 30 mM (about 1.9 g/L) of acetic acid (within the first 20 days) together with the slow establishment of carboxydotrophic activity (over 10 days of CO partial pressures of 20 kPa) and the toxicity of PAC compounds may have hindered methanogens causing their gradual washout in favor of acetogenic Clostridia. Acetic acid (*i.e.*, the undissociated form of acetate) concentrations of 0.3 and 2.4 g/L inhibited specific methanogenic activity by 50% and 90%, respectively, during mesophilic mixed culture fermentations of H_2_/CO_2_ [46]. Similarly, a previous work showed how an increase in CO partial pressure from 0.1 to 0.2 atm at 35 °C induced a fourfold decrease in CO methanation yield, while simultaneously elevating specific CO uptake rates and favoring hydrogenogenesis [44]. Other studies reported methanogenesis inhibition during the co-fermentation of PAC and syngas. At 30 °C and pH 6, methanogenesis was severely inhibited, and acetate, butyrate, and other carboxylic acids up to caproate accumulated in the fermentation broth [18]. Although that system was not optimized for gas fermentation, 46% of the CO fed into the system was metabolized throughout the whole experimental period [18]. In another work, syngas and PAC were co-fermented in shaking flasks under mesophilic and thermophilic conditions. There, lower initial PAC loadings completely inhibited methanogenesis before carboxydotrophic activity and PAC degradation, leading to the accumulation of acetate and other SCCs [48].

Conversely, despite starting under similar conditions to the mesophilic process (in terms of PAC, pH, HRT and gas partial pressures), the thermophilic process produced methane concomitantly with the start of carboxydotrophic activity. The higher favorability of hydrogenogenic reactions at higher temperatures [94], the quick decrease of CO below 10 kPa, and the low undissociated carboxylates concentrations, all contributed to the enrichment of *Methanosarcina* and *Methanothermobacter. Methanosarcina* is a versatile methanogen able to perform acetoclastic, methylotrophic and hydrogenotrophic methanogenesis [95] and may have been responsible for methanogenesis up to the very end of the fermentation. M*ethanothermobacter* species such as *Methanothermobacter thermautotrophicus* or *Methanothermobacter marburgensis* are carboxydotrophic methanogens able to oxidize CO to produce H_2_ and CO_2_ and later convert them to CH_4_ [96]. For instance, *Mb. marburgensis* is able to grow under up to 50 kPa CO and to produce methane and even traces of acetate [97]. Here, *Mb. marburgensis* may have been the major carboxydotrophic microorganism in the thermophilic system up to the enrichment of *Mo. thermoacetica*. *Mo. thermoacetica* is a well-known thermophilic acetogenic microorganism with a versatile metabolism, capable of utilizing various substrates such as sugars [98] as well as CO or H_2_/CO_2_ [99, 100]. Previous studies have indicated its ability to degrade lignin-derived products, including furfural, guaiacol, vanillin, and syringol, ultimately producing acetate [101]. Furthermore, *Mo. thermoacetica* has been reported to possess an inducible CO-dependent O-demethylating capability for the degradation of methylated aromatics, which facilitates the integration of O-methyl groups into the acetyl-CoA pathway [102]. However, no evidence suggests that here *Mo. thermoacetica* participated to PAC components removal. *Thermoanaerobacterium thermosaccharolyticum* is an anaerobic thermophilic bacterium that can ferment cellulose and hemicellulose and other cellulosic sugars into H_2_, acetate, lactate, ethanol, butyrate and butanol. Thermophilic synthetic co-cultures of *T. thermosaccharolyticum* and *Clostridium thermocellum* converted untreated lignocellulose waste into bioethanol [103]. Solventogenic cells of *T. thermosaccharolyticum* were even reported to degrade paraffin oil, a mixture of saturated hydrocarbons, to produce ethanol and butanol [104]. *Thermoanaerobacterium* and *Syntrophaceticus* were the main genera enriched during several thermophilic anaerobic processes at high loads of different intermediates of lignin degradation [105]. Similarly, *Syntrophaceticus* was enriched during the thermophilic degradation of phenyl acids and was considered a primary acetate oxidizer in association with hydrogenotrophic methanogens [106]. Syntrophic acetate oxidizer like *Syntrophaceticus* can convert acetate into H_2_ and CO_2_ via the oxidative Wood–Ljungdahl pathway [107] only at low H_2_ partial pressures, thus forcing syntrophic acetate oxidizers to grow dependently on hydrogenotrophic methanogens [108].

Here, it is possible that *Symbiobacteriales, Thermoanaerobacterium* and other acidogenic microorganisms degraded some PAC components into primarily acetate while *Syntrophaceticus* oxidized the acetate into CO_2_ and H_2_. Then *Methanothermobacter*, *Methanosarcina* and *Methanoculleus* converted acetate and H_2_/CO_2_ into CH_4_. Another work reported similar associations during the thermophilic conversion of phenol into CH_4_ in an anaerobic membrane bioreactor [29]. There, *Clostridium* sensu stricto degraded phenol to acetate via benzoate, while syntrophic acetate oxidizers and *Methanothermobacter* associations were essential to maintain a thermodynamically favorable process. *Syntrophaceticus* and other syntrophic bacteria oxidized the acetate from phenol into CO_2_ and H_2_ while *Methanothermobacter* produced CH_4_ via hydrogenotrophic methanation. An impaired methanogenic population lead to increasing H_2_ partial pressure inhibiting syntrophic acetate oxidizing bacteria and reducing the thermodynamic feasibility of phenol conversion [29]. Here, the accumulation of untreated PAC components or metabolic intermediates of PAC components degradation could have resulted in the inhibition and subsequent wash-out of hydrogenotrophic methanogens such as *Methanosarcina* and *Methanoculleus*, leading to increasing H_2_ partial pressure. Higher H_2_ partial pressure inhibited syntrophic acetate oxidation [108], altering the overall community dynamics and the bioenergetics involved in the degradation of some PAC components. An already weakened *Methanothermobacter* population, compounded by slow CO growth kinetics [96] and increasing toxicant concentrations in the fermentation broth, might have resulted in the abrupt decrease of carboxydotrophic activity.

The enrichment of *Corynebacterium* and *Acinetobacter* may suggest for some occasional air intrusions. Even though, *Corynebacteria* and *Acinetobacter* have been reported to be mainly active in aerobic environments, they can grow also in anaerobic ones [87, 109–114]. In another work, low air contamination below detection limit (but quantified to a daily contamination rate of 220 ± 33 mLO_2_/L/d) provided competitive advantage to Actinobacteria and Coriobacteriia over the anaerobic community members but did not completely inhibit methanogenesis during lactate and H_2_/CO_2_ elongation to medium-chain carboxylates [115]. Similarly, minimal air exposure (5–8% in the reactor headspace) did not affect methanogenesis during the thermophilic anaerobic digestion or switch grass [116]. Facultative anaerobic and aerobic microorganisms can consume the O_2_ in the air contamination limiting the strictly anaerobic members of the community to O_2_ exposure [117]. The efficacy of this protective mechanism is affected on the composition of the microbial community and the extent of oxygen contamination [118]. Here, *Corynebacterium* and *Acinetobacter* may have consumed the oxygen available and possibly mitigated the effects on the bioreactor performances. Despite their enrichment, no significant correlation emerged between low CO uptake rates and the presence of *Corynebacterium* and *Acinetobacter*. Occasional air contamination may have contributed to further weaken the anaerobic microorganisms (already inhibited by PAC components) but there is no evidence that it was the primary cause of lower CO conversion rates. On the other hand, the ability of *Corynebacterium* and *Acinetobacter* to degrade aromatic compounds [119–126] may have improved overall reactor performances. Some *Acinetobacter* spp. possess genes encoding a CO dehydrogenase and can grow on CO, although aerobically, as sole carbon and energy source [127, 128], but there is no experimental evidence in this work.

There are numerous documented instances of PAC component degradation during anaerobic digestion of PAC, albeit with varying removal rates and COD recoveries [29, 129–131]. These differences can be attributed to the composition of PAC since the degradation of specific PAC components can be significantly influenced by the presence of other toxic compounds [132–135]. Nevertheless, some works have attempted to elucidate the degradation pathways of PAC components such as phenol, furfural guaiacol and cresol reporting for the production of short chain carboxylates or methane. For instance, benzoyl-CoA was reported to be a central intermediate during the anaerobic degradation of phenol via 4-hydroxybenzoate. Benzoyl-CoA is subsequently converted via β-oxidation ring opening into three molecules of acetyl-CoA, which are further transformed into acetate [30, 31]. Furfural was reported to be converted into furoic acid via furfuryl alcohol, ultimately leading to the production of acetate [30]. Similarly, the anaerobic degradation of guaiacol generates acetate via demethylation of guaiacol to catechol [101]. The anaerobic degradability of cresols, on the other hand, depends on the position of the hydroxyl group. For example, *m-*cresol, is generally considered the most recalcitrant to anaerobic degradation [17]. Nonetheless, during *m-*cresol degradation, fumarate is added to the methyl group of *m-*cresol to form 3-hydroxybenzyl succinate. Activation and β-oxidation lead to succinyl-CoA and 3-hydroxybenzoyl-CoA [133].

### Process stability and re-inoculations

The carboxydotrophic activity in thermophilic system, as for the mesophilic one, was influenced by the interplay of factors such as PAC loading, biomass concentration and microbial diversity. Temperature likely played a critical role determining the stability of syngas and PAC co-fermentation. Some works assessing the effects of temperature on the anaerobic digestion of phenolic compounds or of the aqueous phase generated from hydrothermal liquefaction of cornstalk reported higher removal of aromatic compounds at mesophilic conditions and accumulation of untreated compounds at thermophilic conditions [129, 136]. Other factors such as inoculum origin and diversity, process operations and reactor design play critical roles in the successful establishment of functional microbial cultures for wastewater detoxification [137]. Here, PAC loading potentially led to diminished functionality and diversity within both reactor microbiomes, increasing the toxicant level and resulting ultimately in the decline of CO conversion rates. Anaerobic carboxydotrophic microorganisms rely on carbon monoxide dehydrogenase to catalyze CO conversion into H_2_ and CO_2_ [138]. This may render CO uptake a rather fragile process when exposed to toxic and very complex wastewaters such as PAC.

The selective pressure exerted by toxic components or other process parameters could enhance tolerance, reducing adaptation time and improving the biodegradation capabilities of the enriched culture [126]. However, a diminishing community richness poses a risk of losing critical functionality vital for the success of the process [77]. Even a minor alteration in a crucial parameter may inhibit a highly specialized microbial consortium [139]. Similarly to bioaugmentation, a strategy commonly employed to recover inhibited anaerobic digestion and other bioprocesses [140–145], re-inoculating the reactors resulted in high removal efficacies of PAC components and in sustained carboxydotrophic activity even under higher PAC loads. Re-inoculations bolstered both biomass concentration and microbial diversity, allowing for quick recovery (consistently within one day) and extending significantly the process time. Another work proved how co-digesting PAC and manure (as source of organics and active cells) improved both methane yields and the maximum PAC loading by diluting toxic compounds [146]. Here, higher multifunctional activity persisted for over 50 and 90 days for the mesophilic and the thermophilic process, respectively, up to 6% v/v (0.8 gCOD/L/d). This PAC loading level was the maximum achievable, maintaining an average VSS concentration of approximately 3 g/L.

Cell retention systems or immobilization technologies may offer alternative approaches for retaining microorganisms within the system. Cell retention, for instance, is a technology employed to improve cell concentrations by preventing cell washout, especially during continuous bioprocesses characterized by low cell densities, such as anaerobic syngas fermentation [147, 148]. Alternatively, packed reactors have been also employed for mixed cultures syngas fermentation processes [149, 150]. Numerous studies have highlighted the advantageous effects of biochar as amendment and packing material during the anaerobic digestion of PAC. There, biochar provided structural support for microbial growth and facilitated interactions among microorganisms, thereby enhancing process performance [5, 6, 17, 19–28, 32, 146, 151, 152].

### L-Malate production

Both mesophilic and thermophilic mixed cultures degraded PAC components to a level that allowed *A. oryzae* to grow (up to PAC loading of 6 v/v %) and to convert SCCs into fungal biomass and L-malate. Among the compounds contained in the bio-oil generated from the pyrolysis of wheat straw, phenol, furfural, guaiacol, 2-cyclopentenone and cresol were severely inhibiting the growth and L-malate production of *A. oryzae* [58]. In a previous work, 2.5 v/v % of the same PAC as used in this study proved to be inhibitory and impeded the growth of *A. oryzae* [48]. Here, the growth of *A. oryzae* was minimal and none in two flasks only with the effluent from the mesophilic process collected on day 116 (4% v/v) and day 213, respectively. These results were likely linked to the accumulation of untreated PAC components, or accumulation of by-products from the degradation of PAC components, as the decreasing e-mol recovery indicated.

Even though process optimization was not the scope of this work, the highest yields achieved in this study are similar to what was described in other works. When grown in shaking flasks on acetate, *A. oryzae* yielded up to 21% g_L-malate_/g_acetate_ but the production was highly dependent upon the initial acetate concentration [64]. In bioreactor experiments optimized for L-malate production from acetate, *A. oryzae* produced about 29 g/L L-malate corresponding to a 29% g_L-malate_/g_acetate_ L-malate yield [63]. During the cultivation of *A. oryzae* with acetate and acetol from a detoxified PAC, L-malate was produced up to 7.3 ± 0.3 g/L (corresponding to a yield of 20 ± 0.01 g_L-malate_/g_substrates_) [69]. In a sequential syngas to L-malate fermentation process (with *Cl. ljungdahlii* and *A. oryzae* as microbial catalysts), the fermentation medium rich in acetate from stage one was fed directly to *A. oryzae*. L-Malate production with acetate from the syngas fermentation as sole carbon source reached yields of 28 w/w %. The presence of macro- and micronutrients in the fermentation broth from the first stage syngas fermentation was highlighted to have major positive effects on *A. oryzae* growth and improved L-malate yields [50]. Similar synergies may have occurred here.

## Conclusions

This study demonstrates the ability of mesophilic and thermophilic mixed cultures to recover carbon and energy simultaneously from syngas sequestration and PAC components degradation into CH_4_, acetate and other short-chain carboxylates. The carboxylates generated during syngas and PAC co-fermentation were subsequently converted to L-malate by *Aspergillus oryzae* in a second-stage fermentation, increasing the overall process selectivity. The findings highlight the diversity of process regimes that can be achieved by simply changing the operating temperature. The mesophilic process was stable, non-methanogenic and short-chain carboxylates accumulated in the medium. The enrichment of *Caproiciproducens* suggests the potential of the mesophilic process for the production of medium-chain carboxylates. Conversely, the thermophilic process converted syngas and PAC into primarily methane but suffered from unstable CO conversion, potentially due to unfavorable process conditions. The instability was addressed through the regular injection of fresh inoculum. Integrating animal manure as a substrate during thermophilic conversion of syngas and PAC may resolve possible instability.

This work represents a successful effort in demonstrating the potential of a two-stage process for producing platform chemicals from gaseous and toxic substrates. It identifies critical parameters essential for the co-fermentation of syngas and PAC, thereby laying the groundwork for further advancements in this field. Key areas requiring attention include the optimization of operational parameters, bioreactor design, and the implementation of a two-stage continuous fermentation with particular focus on syngas and PAC flows based on a real pyrolysis process. Addressing these aspects will be crucial in advancing the readiness of this technology for practical applications.

### Supplementary Information

Supplementary material 1: Table S1. GC–MS characterization of the aqueous condensate deriving from the fast pyrolysis of Miscanthus was performed by the Thünen Institute of Wood Research, (Hamburg, Germany). Table S2. Composition in gCOD/Ld and electron moles (e-mM/d) of the feed (for both syngas and PAC) for both the mesophilic and the thermophilic semi-continuous fermentations. Table S3. Conversion factors for electron balances. Figure S1. Fermentation profile of the mesophilic process. Top x-axis shows increases in PAC loading, bottom x-axis shows the elapsed fermentation time. The red bar indicate the period of weekly re-inoculations. (a) pH and redox potential. (b) Partial pressures of CO and H2. (c) Concentration of undissociated carboxylates. (d) Removal efficacy of each cresol isomer. Negative values indicate production. (e) Concentrations of total (TSS) and volatile suspended solids (VSS). Figure S2. Fermentation profile of the thermophilic process. Top x-axis shows increases in PAC loading, bottom x-axis shows the elapsed fermentation time. Red arrows point to re-inoculation events, the red bar indicate the period of weekly re-inoculations. (a) pH and redox potential. (b) Partial pressures of CO and H2. (c) Concentration of undissociated carboxylates. (d) Removal efficacy of each cresol isomer. Negative values indicate production. (e) Concentrations of total and volatile suspended solids. (f) Relative abundance of the enriched archaeal genera (based on mcrA gene amplicon sequencing variants). Others include all microbial genera with abundance lower than 1%. Figure S3. Spearman’s rank correlations between relative abundance of methanogens (based on mcrA gene amplicon sequencing variants) and process parameters for the thermophilic semi-continuous STR enrichment. The strength of the correlation is represented by the size of the circle and intensity of the color. Blue circles indicate positive correlations. Red circles indicate negative correlations. p values are shown for non-significant correlations (p < 0.05). Figure S4. CO, H2, CH4 production rates during the decrease in CO uptake rates for the thermophilic syngas and PAC co-fermentation. Figure S5. Photos depicting *A. oryzae* growth for all aerobic flask fermentations after 72 h. The pictures labeled with 6% PAC were recorded from cultivations with the supernatant collected after 207 days of fermentation for both mesophilic and thermophilic process. Pictures labeled with 6% PAC END were recorded from cultivations with the supernatant collected after 213 days of fermentation for both mesophilic and thermophilic process. Table S4. L-malate and SCCs concentrations over time and L-malate highest yields per SCCs consumed. Numbers are mean values with standard deviations calculated from three replicates. The medium was the supernatant of the fermentation broth collected from the mesophilic reactor. Figure S6. L-malate, acetate, propionate and butyrate concentrations over time. Numbers are mean values with standard deviations calculated from three replicates. The medium was the supernatant of the fermentation broth collected from the mesophilic reactor. Table S5. L-malate and SCCs concentrations over time and L-malate highest yields per SCCs consumed. Numbers are mean values with standard deviations calculated from three replicates. The medium was the supernatant of the fermentation broth collected from the thermophilic reactor. Figure S7. L-malate, acetate, propionate and butyrate concentrations over time. Numbers are mean values with standard deviations calculated from three replicates. The medium was the supernatant of the fermentation broth collected from the thermophilic reactor.

## Data Availability

The 16 s rRNA and mrcA raw sequence data without adapters used in this study have been deposited in the European Nucleotide Archive (ENA) under the study accession number PRJEB72504 (http://www.ebi.ac.uk/ena/data/view/PRJEB72504). Further datasets used and/or analyzed during the current study are available from the corresponding author on reasonable request.
