# Supplementary material for: Two-stage conversion of syngas and pyrolysis aqueous condensate into L-malate
Source: Biotechnol Biofuels Bioprod. 2024 Jun 21;17:85. doi: 10.1186/s13068-024-02532-2 (PMC11191387; doi:10.1186/s13068-024-02532-2)
Supplement: Supplementary file 1 — Supplementary material 1: Table S1. GC–MS characterization of the aqueous condensate deriving from the fast pyrolysis of Miscanthus was performed by the Thünen Institute of Wood Research, (Hamburg, Germany). Table S2. Composition in gCOD/Ld and electron moles (e-mM/d) of the feed (for both syngas and PAC) for both the mesophilic and the thermophilic semi-continuous fermentations. Table S3. Conversion factors for electron balances. Figure S1. Fermentation profile of the mesophilic process. Top x-axis shows increases in PAC loading, bottom x-axis shows the elapsed fermentation time. The red bar indicate the period of weekly re-inoculations. (a) pH and redox potential. (b) Partial pressures of CO and H2. (c) Concentration of undissociated carboxylates. (d) Removal efficacy of each cresol isomer. Negative values indicate production. (e) Concentrations of total (TSS) and volatile suspended solids (VSS). Figure S2. Fermentation profile of the thermophilic process. Top x-axis shows increases in PAC loading, bottom x-axis shows the elapsed fermentation time. Red arrows point to re-inoculation events, the red bar indicate the period of weekly re-inoculations. (a) pH and redox potential. (b) Partial pressures of CO and H2. (c) Concentration of undissociated carboxylates. (d) Removal efficacy of each cresol isomer. Negative values indicate production. (e) Concentrations of total and volatile suspended solids. (f) Relative abundance of the enriched archaeal genera (based on mcrA gene amplicon sequencing variants). Others include all microbial genera with abundance lower than 1%. Figure S3. Spearman’s rank correlations between relative abundance of methanogens (based on mcrA gene amplicon sequencing variants) and process parameters for the thermophilic semi-continuous STR enrichment. The strength of the correlation is represented by the size of the circle and intensity of the color. Blue circles indicate positive correlations. Red circles indicate negative correlations. p values [file 13068_2024_2532_MOESM1_ESM.pdf]

Two-stage conversion of pyrolysis syngas and pyrolysis aqueous condensate into L-malate

Alberto Robazza[a], Flávio C. F. Baleeiro[b], Sabine Kleinsteuber[b]; Anke Neumann[a],

[a] Institute of Process Engineering in Life Sciences 2: Electro Biotechnology, Karlsruhe Institute of Technology - KIT, 76131 Karlsruhe, Germany;

[2] Department of Microbial Biotechnology, Helmholtz Centre for Environmental Research - UFZ, 04318 Leipzig, Germany

Characterization of the *Miscanthus* Pyrolysis Aqueous Condensate

Table S1. GC-MS characterization of the aqueous condensate deriving from the fast pyrolysis of Miscanthus was performed by the Thünen Institute of Wood Research, (Hamburg, Germany).

Compound	wt.% wet	
	Average	St.Dev.
Water Content	81.700	1.744
Acetic acid	3.406	0.790
Propionic acid	0.508	0.080
Butyric acid	0.050	0.000
Acetic acid 2-hydroxyethyl ester	0.040	0.004
Propanoic acid methyl ester	0.013	0.007
Ethylene glycol	1.541	0.861
2-Propen-1-ol (NIST MQ 84)	0.050	0.010
Acetaldehyde, hydroxy-	0.496	0.079
Propionaldehyde, 3-hydroxy	0.087	0.007
Butyraldehyde	0.018	0.002
Crotonaldehyde, cis	0.156	0.004
Crotonaldehyde, trans	0.043	0.004
2-Butenal, 2-methyl- (NIST MQ 92)	0.011	0.002
poss: 2-Pentenal, (E)- (NIST MQ 89)	0.012	0.002
Butanedial or Propanal (NIST MQ 88)	0.037	0.015
Acetol (Hydroxypropanone)	2.704	0.616
Acetonylacetone (Hexandione, 2,5-)	0.006	
Butanone, 2-	0.128	0.006
Butanone, 1-hydroxy-2-	0.288	0.042
Butandione, 2,3- (Diacetyl)	0.364	0.012
Acetoin (Hydroxy-2-butanone, 3-)	0.041	0.009
Propan-2-one, 1-acetyloxy-	0.062	0.020
Cyclopentanone	0.083	0.003
Cyclopenten-1-one, 2-	0.145	0.029
Cyclopenten-1-one, 2,3-dimethyl-2-	0.009	0.003
Cyclopenten-1-one, 2-methyl-2-	0.051	0.009
Cyclopenten-1-one, 3-methyl-2-	0.022	0.004
Cyclopenten-1-one, 2-hydroxy-2-	0.010	0.002
Cyclopenten-3-one, 2-hydroxy-1-methyl-1-	0.062	0.007
Cyclohexen-1-one, 2-	0.004	0.001
Methyl vinyl ketone = 2-Butenone (NIST MQ 90)	0.013	0.004
poss: 2-Butanone, 3-methyl- (NIST MQ 88)	0.031	0.006
3-Buten-2-one, 3-methyl- (NIST MQ 88)	0.031	0.002
2,3-Pentanedione	0.068	0.007
3-Penten-2-one (NIST MQ 84)	0.036	0.004

2-Butanone, 4-hydroxy- (NIST MQ 84)	0.021	0.003
poss: 2-Pentanone, 4-hydroxy- (NIST MQ 82)	0.017	0.004
Isomere of 2-Cyclopenten-1-one, 3-methyl-	0.011	0.002
2-Butanone, 1-hydroxy-3-methyl- (NIST MQ 78)	0.009	0.002
2-Cyclopenten-1-one, 3,4-dimethyl-	0.013	0.002
4-Cyclopentene-1,3-dione (NIST MQ 86)	0.009	
2-Cyclopenten-1-one, 2,3,4-trimethyl- (NIST MQ 88)	0.005	0.002
Isomere of Cyclopenten-1-one, 2,3-dimethyl-2-	0.020	
Furanone, 2(5H)-	0.073	0.020
Furaldehyde, 2-	0.399	0.067
Furaldehyde, 3-	0.026	0.006
Furaldehyde, 5-methyl-2-	0.018	0.005
Ethanone, 1-(2-furanyl)-	0.021	0.003
Furan-2-one, 5-methyl-, (5H)-	0.018	0.001
Furan-2-one, 3-methyl-, (5H)-	0.016	0.008
Furan-2-one, 2,5-dihydro-3,5-dimethyl-	0.023	0.007
Butyrolactone, γ-	0.030	0.008
Furan, tetrahydro-2-methoxy- (NIST MQ (/)	0.005	
Furan-2-one, 4-methyl-(5H)- (NIST MQ 88)	0.008	0.004
Benzene, 1-methoxy-3-methyl-	0.003	
Benzene, 1-methoxy-4-methyl-	0.006	
Benzaldehyde	0.005	0.001
poss: Benzaldehyde, 2-hydroxy-	0.008	0.002
Phenol	0.056	0.010
Cresol, o-	0.028	0.005
Cresol, p-	0.027	0.005
Cresol, m-	0.016	0.002
Phenol, 2,5-dimethyl-	0.007	0.000
Phenol, 2,4-dimethyl-	0.005	0.002
Phenol, 2,6-dimethyl-	0.004	0.001
Phenol, 4-ethyl-	0.036	0.013
Phenol, ethyl-methyl-	0.004	0.001
Guaiacol	0.072	0.016
Guaiacol, 4-methyl-	0.033	0.009
Guaiacol, 4-ethyl-	0.015	0.004
Guaiacol, 4-allyl- (Eugenol)	0.007	0.002
Guaiacol, 4-propyl-	0.002	0.001
Guaiacol, 4-propenyl- cis (Isoeugenol)	0.007	0.003
Vanillin	0.019	
Syringol	0.010	0.003
Syringol, 4-methyl-	0.003	0.001
2-Acetyl-5-norbornene (NIST MQ 92)	0.005	0.001
1,3-Dioxolane, 2-methyl- (NIST MQ 62)	0.019	0.007
1,3-Dioxolane, 2-ethyl-	0.003	0.001
poss: 1,4-Dioxin, 2,3-dihydro-	0.012	0.003
2,2'-Bi-1,3-dioxolane (NIST MQ 87)	0.018	0.003

Fermentation medium

The modified BA medium was composed as follows. For each liter of medium added: 100 mL of mineral salt solution, 800 mL of phosphate buffer solution, 10 mL of vitamin solution, 10 mL of trace elements solution, 5 mL of resazurin solution and 3 mL of reducing agent solution. The mineral salt solution was prepared with the following salt concentrations: NH_4Cl, 161.2 g/L; $MgCl_2 \times 6H_2O$, 5.4 g/L; $CaCl_2 \times 2H_2O$ 6.5 g/L, NaCl, 30 g/L. The phosphate buffer solution was prepared with 136 g/L KH_2PO_4. The vitamin solution was composed of: biotin, 0.002 g/L; folic acid, 0.002 g/L; pyridoxin, 0.01 g/L; thiamin, 0.005 g/L; riboflavin, 0.005 g/L; nicotinic acid, 0.005 g/L; Ca-pantothenate, 0.005 g/L; vitamin B12, 0.005 g/L; aminobenzoic acid, 0.005 g/L; liponic acid, 0.005 g/L). The trace elements solution contained the following compounds: $FeCl_2 \times 4H_2O$, 1.5 g/L; $MnCl_2$, 0.1 g/L; $CoCl_2 \times 6H_2O$, 0.19 g/L; ZnCl2, 0.07 g/L;

$CuCl2 \times 2H_2O$, 0.002 g/L; $NiCl2 \times 6H_2O$, 0.024 g/L; $Na_2MoO_4 \times 2H_2O$, 0.036 g/L; H_3BO_3, 0.006 g/L; $Na_2SeO_3 \times 5H_2O$, 0.003 g/L; $Na_2WO_4 \times 2H_2O$, 0.02 g/L. The reducing agent solution contained 100 g/L of L-cysteine. The resazurin solution contained 1 g/L resazurin sodium salt. All reagent-grade chemicals were purchased from Sigma-Aldrich (Schnelldorf, Germany) or Carl Roth (Karlsruhe, Germany).

Syngas and PAC load composition

Table S2. Composition in gCOD/Ld and electron moles (e-mM/d) of the feed (for both syngas and PAC) for both the mesophilic and the thermophilic semi-continuous fermentations.

PAC load	1 % v/v	2 % v/v	3 % v/v	4 % v/v	5 % v/v	6 % v/v
$gCOD_{PAC}$/Ld	0.13	0.25	0.38	0.51	0.63	0.76
$gCOD_{Syngas}$/Ld	2.6	2.6	2.6	2.6	2.6	2.6
$gCOD_{Total}$/Ld	2.73	2.85	3.0	3.1	3.2	3.3
e-mol_{PAC}/Ld	15.8	31.7	47.5	63.3	79.1	95.0
e-mol_{Syngas}/Ld	326.3	326.3	326.3	326.3	326.3	326.3
e-$mol_{Substrates}$/Ld	342.1	358.0	373.8	389.6	405.5	421.3
PAC/syngas [%]	4.6	9.7	14.6	19.4	24.3	29.1

Equations

Estimation of VSS concentration

Between each re-inoculation event, the concentration of volatile suspended solids (VSS) at time t=j was determined as described by Eq. 1.

$$VSS_{Est,j} = TSS_j - TFS_{Res,j} \text{ [g/L]} \qquad \text{Eq.1}$$

Where TSS_j was determined experimentally at time t=j. As mentioned in the materials and methods section, the weight of the dried pellet was measured and assumed to be representative of the suspended fraction (TSS) of the total solids (TS). The residual amount of the total fixed solids ($TFS_{Ref,j}$) was calculated using the following equation:

$$TFS_{Res,j} = TFS_{Res,j-1} - TFS_{Res,j-1} * \frac{1}{HRT} \text{ [g/L]} \qquad \text{Eq.2}$$

$TFS_{Res,j-1}$ is the residual amount of the total fixed solids determined at time t=j-1. HRT is the hydraulic retention time and was HRT = 20 d in this study. The initial TFS was determined by multiplying the TFS of the inoculum by the dilution factor of the inoculation.

PAC components removal

The concentration of each selected PAC component in the fermentation medium was determined via HPLC analysis of a solution composed of BA medium with PAC concentrations similar to those reported in Supp. Table 2. The removal efficacy of a selected PAC component *i* at time t=j was calculated with the following equation:

$$Removal\ Efficacy_{m,j} = \frac{C_{m,j}}{C_{Th.Broth,m,j}} * 100 \text{ [\%]} \qquad \text{Eq.3}$$

Where $C_{m,j}$ is the concentration of compound i at time t=j determined experimentally via HPLC and $C_{Th.Broth,m,j}$ is the theoretical concentration of the compound i at time t=j in the fermentation broth considering an accumulation in an abiotic system, as shown in equation Eq.4

$$C_{Th.Broth,i,j} = C_{Th.Broth,i,j-1} + d * (C_{Feed,i} - C_{Th.Broth,i,j-1}) \text{ [g/L]} \qquad \text{Eq.4}$$

Where $C_{Th.Broth,m,j}$ is the theoretical concentration of the compound i at time t=j-1; $C_{Feed,m}$ is the determined concentration of the compound i in the feed and d is the dilution rate of the system. The volume of the broth is considered constant and equal to 1.5 L and constant are considered to be also the daily feed and removal (0.075 L/d) of the fermentation broth.

E-mol balances

The daily e-mol recovery was calculated as described by Eq. 5 to determine the daily load of e-mol from PAC.

$$e\text{-}mol\ Recovery_j = \frac{\sum e\text{-}mol_{Products}}{\sum e\text{-}mol_{Substrates}} * 100\ [\%] \qquad \text{Eq.5}$$

The $\sum$e-$mol_{Substrates}$ is the sum of the daily e-mol from syngas and from PAC per broth volume fed into the bioreactors. The volume of the broth is considered constant and equal to 1.5 L. Hydrogen is considered a substrate only when it was consumed, otherwise a product and was accounted as such.

$$e\text{-}\dot{m}ol_{Syngas} = \dot{n}_{CO} * eeq_{CO} + \dot{n}_{H_2} * eeq_{H_2}\ \text{[e-M/d] Eq.6}$$

$$e\text{-}\dot{m}ol_{PAC} = \frac{\dot{m}_{COD,PAC}}{eeq_{PAC}}\ \text{[e-M/d]Eq.7}$$

Where $\dot{n}$ of CO and H_2 are the daily uptake rates determined as described in another work [1]. $\dot{m}_{COD,PAC}$ is the daily flow rate of gCOD of PAC in the feed. We assumed that 1 mol of electron equivalents is equal to 8 gCOD. Considering that the COD (Chemical oxygen demand) is the oxygen required to completely oxidize the carbonaceous fraction of organic compounds and that 1 eeq. is released upon complete oxidation of carbonaceous compounds, then from the half reaction RS1, is can assumed that 1/4 mol of O2 (8 g) would be consumed in accepting the 1 e-mol.

$$1/2\, H_2O = \ 1/4\, O_2 + H^+ + e^- \qquad \text{RS1}$$

The $\sum$e-$mol_{Products}$ the sum of the daily production rate in e-mol of methane, H_2 (when produced), formate, acetate, ethanol, propionate and butyrate. It was calculated as described in equation Eq.8. Negative productivities were not accounted.

$$e\text{-}\dot{m}ol_{i,j} = e\text{-}M_{i,j-1} * d + e\text{-}M_{i,j} - e\text{-}M_{i,j-1}\ \text{Eq.8}$$

For compounds like acetate and propionate, which have a significant concentration in the PAC, the daily acetate e-mol feed was subtracted to the value calculated with Eq.8

Table S3. Conversion factors for electron balances.

Compound	Chemical Formula	Molecular Weight	mol e^- /mol
Formate	CH_2O_2	46.1	2.0
Acetate	$C_2H_4O_2$	60.0	8.0
Propionate	$C_3H_6O_2$	74.0	14.0
Butyrate	$C_4H_8O_2$	88.1	20.0
Ethanol	C_2H_6O	46.0	12.0
Hydrogen	H_2	2.0	2.0
Carbon monoxide	CO	28.0	2.0
Carbon dioxide	CO_2	44.0	0.0
Methane	CH_4	16.0	8.0

Mesophilic process

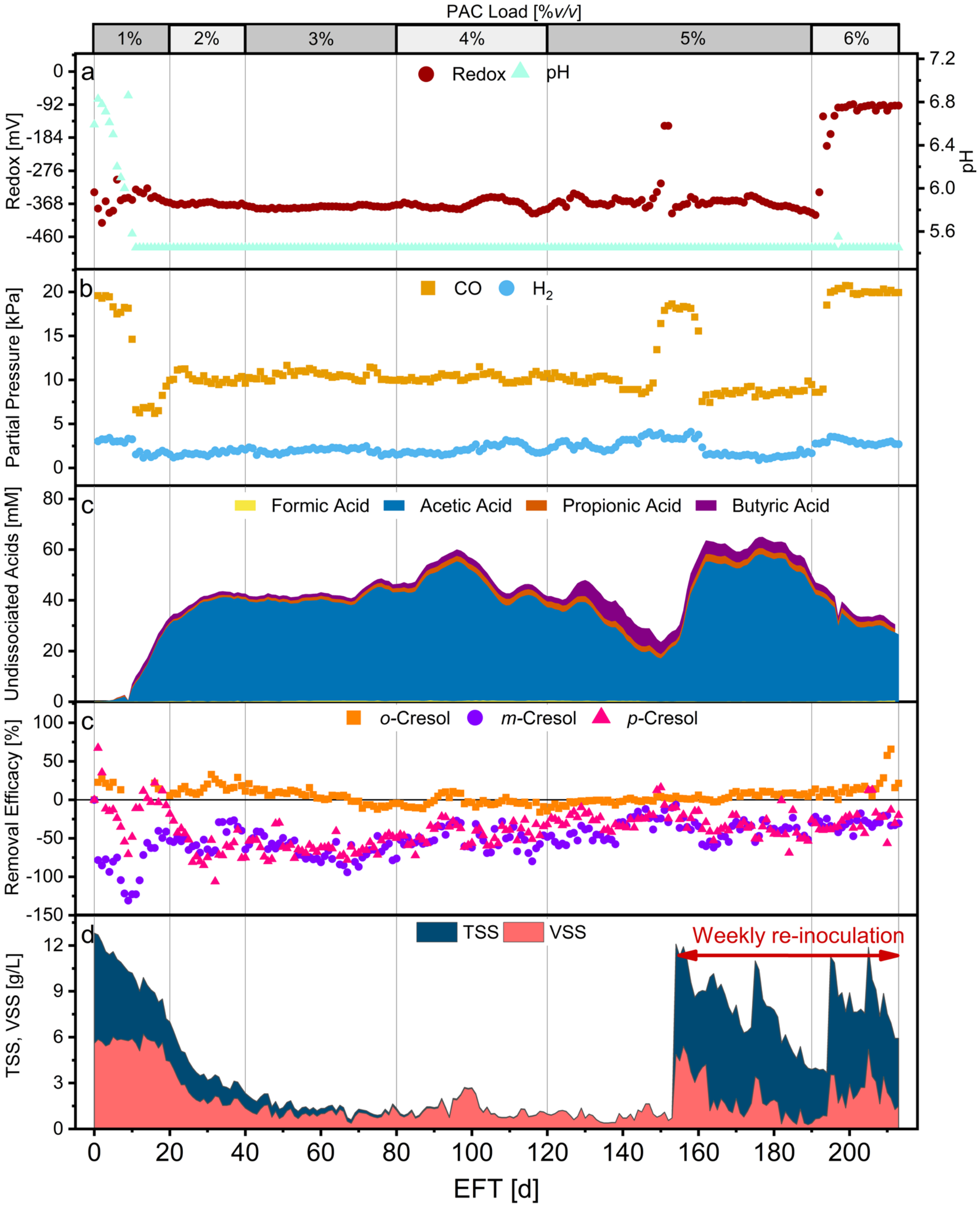

Figure S1. Fermentation profile of the mesophilic process. Top x-axis shows increases in PAC loading, bottom x-axis shows the elapsed fermentation time. The red bar indicate the period of weekly re-inoculations. (a) pH and redox potential. (b) Partial pressures of CO and H_2. (c) Concentration of undissociated carboxylates. (d) Removal efficacy of each cresol isomer. Negative values indicate production. (e) Concentrations of total (TSS) and volatile suspended solids (VSS).

Thermophilic process

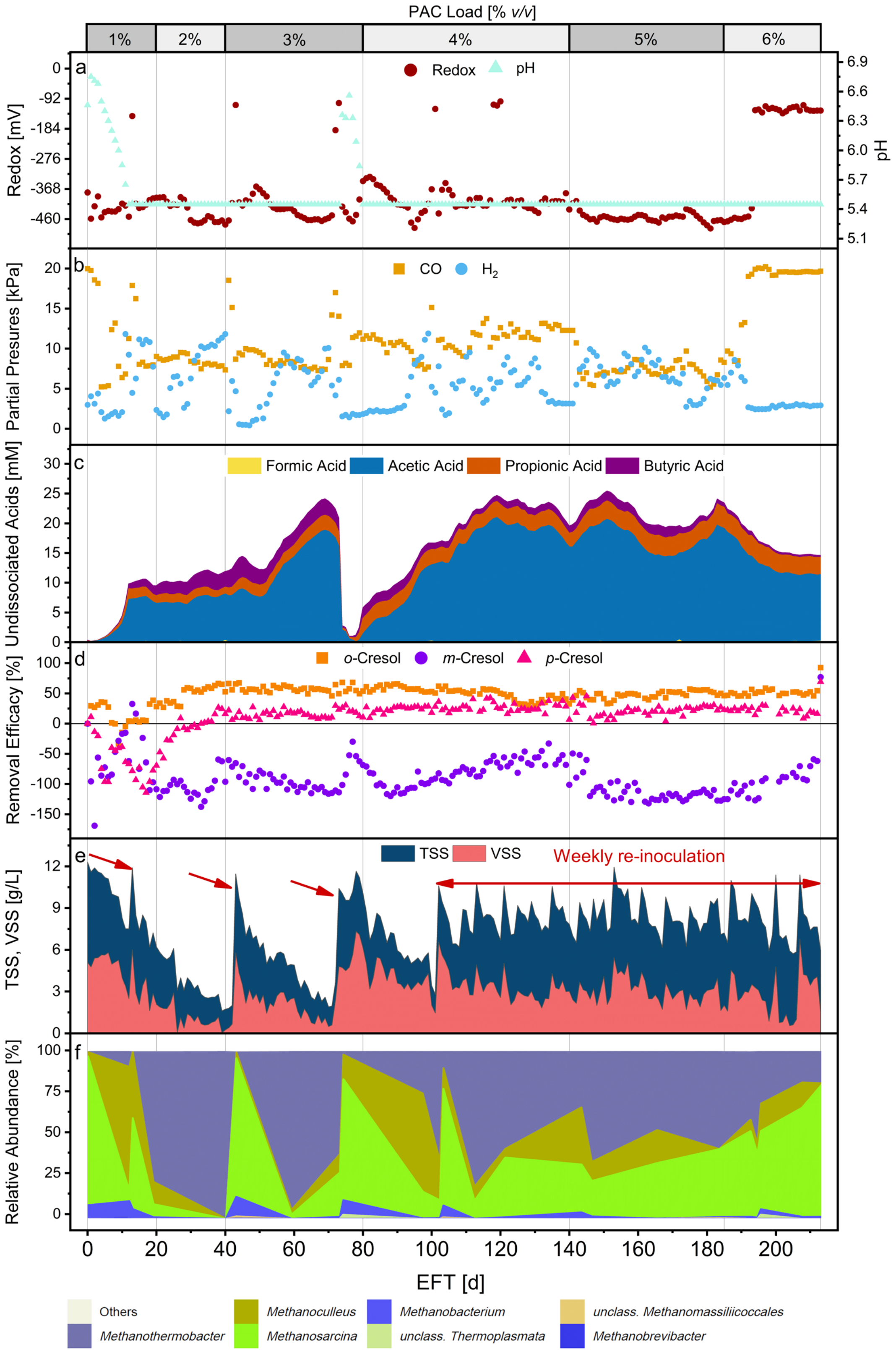

Figure S2. Fermentation profile of the thermophilic process. Top x-axis shows increases in PAC loading, bottom x-axis shows the elapsed fermentation time. Red arrows point to re-inoculation events, the red bar indicate the period of weekly re-inoculations. (a) pH and redox potential. (b) Partial pressures of CO and H_2. (c) Concentration of undissociated carboxylates. (d) Removal efficacy of each cresol isomer. Negative values indicate production. (e) Concentrations of total and volatile suspended solids. (f) Relative abundance of the

enriched archaeal genera (based on mcrA gene amplicon sequencing variants). Others include all microbial genera with abundance lower than 1%.

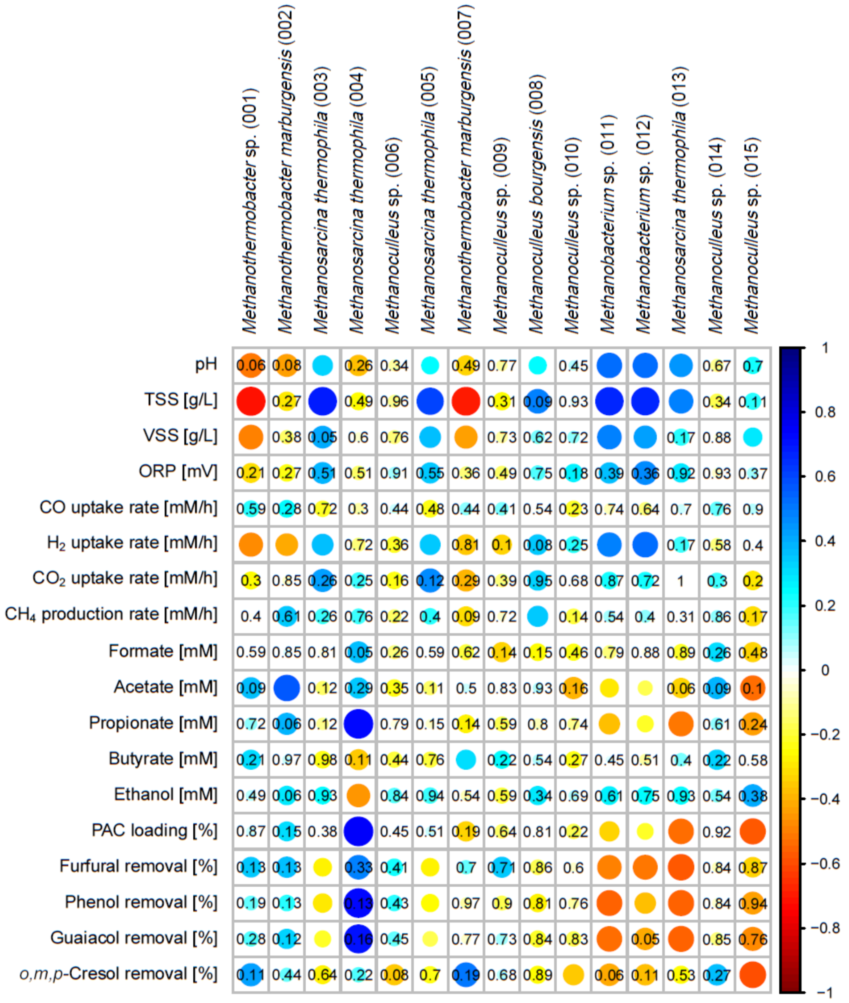

Figure S3. Spearman's rank correlations between relative abundance of methanogens (based on mcrA gene amplicon sequencing variants) and process parameters for the thermophilic semi-continuous STR enrichment. The strength of the correlation is represented by the size of the circle and intensity of the color. Blue circles indicate positive correlations. Red circles indicate negative correlations. p values are shown for non-significant correlations ($p<0.05$).

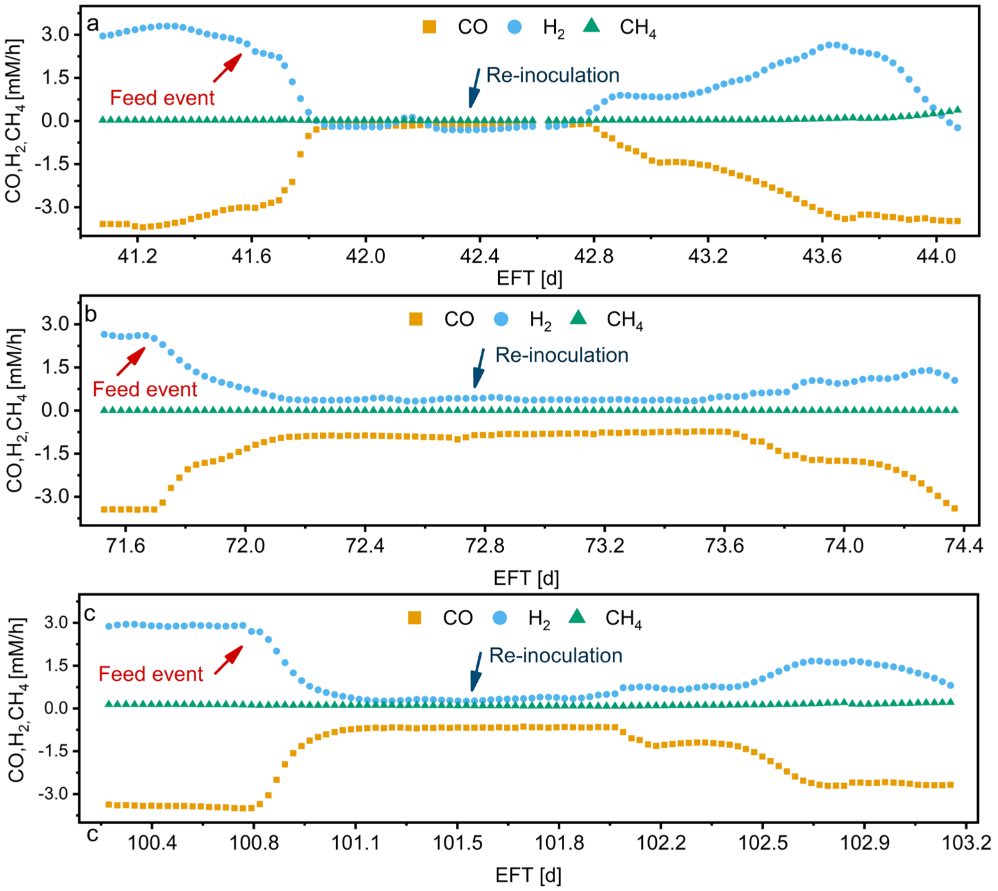

Figure S4. CO, H_2, CH_4 production rates during the decrease in CO uptake rates for the thermophilic syngas and PAC co-fermentation.

Growth of *Aspergillus oryzae* in reactor effluent

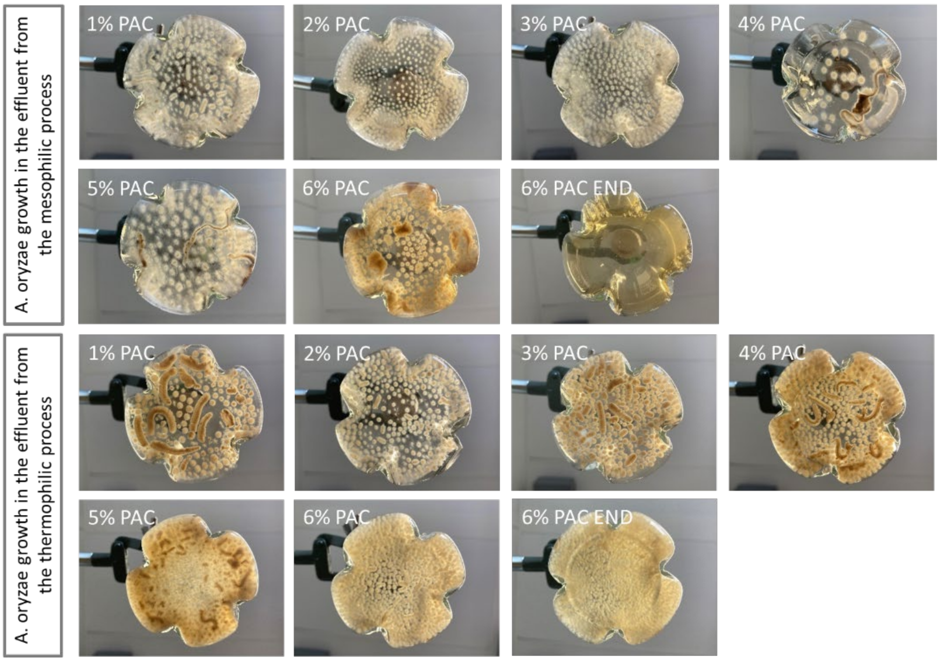

Figure S5. Photos depicting A. oryzae growth for all aerobic flask fermentations after 72 hours. The pictures labelled with 6% PAC were recorded from cultivations with the supernatant collected after 207 days of fermentation for both mesophilic and thermophilic process. Pictures labelled with 6% PAC END were recorded from cultivations with the supernatant collected after 213 days of fermentation for both mesophilic and thermophilic process.

Table S4. L-malate and SCCs concentrations over time and L-malate highest yields per SCCs consumed. Numbers are mean values with standard deviations calculated from three replicates. The medium was the supernatant of the fermentation broth collected from the mesophilic reactor.

Collection time from reactor	PAC load [% v/v]	Hours from *A. oryzae* inoculation [h]	0	24	48	72	96
20 days	1% PAC	L-malate [mM]	0.0	0.0	2.1 ± 0.7	18.4 ± 0.5	11.9 ± 1.8
		SCCs [mM]	209.4 ± 5.5	180.4 ± 2.4	161.9 ± 3.7	120.9 ± 3.5	0.0
40 days	2% PAC	L-malate [mM]	0.0	0.0	3.7 ± 2.1	16.7 ± 1.2	20.5 ± 2.1
		SCCs [mM]	218.4 ± 0.8	235.7 ± 4.2	211.8 ± 7.3	168.2 ± 3.2	0.0
80 days	3% PAC	L-malate [mM]	0.0	0.0	5.8 ± 0.4	28.6 ± 3.7	33.0 ± 0.8
		SCCs [mM]	234.5 ± 2.7	224.1 ± 3.1	206.8 ± 1.7	165.4 ± 4.5	2.7 ± 0.1
116 days	4% PAC	L-malate [mM]	0.0	0.0	0.6 ± 0.1	4.3 ± 0.8	14.4 ± 1.9
		SCCs [mM]	252.0 ± 4.6	242.6 ± 5.1	248.1 ± 0.7	257.0 ± 2.2	196.4 ± 2.2
190 days	5% PAC	L-malate [mM]	0.0	0.0	0.4 ± 0.1	8.2 ± 3.9	28.8 ± 1.7
		SCCs [mM]	303.6 ± 0.8	307.9 ± 3.6	306.1 ± 3.4	310.9 ± 2.6	12.8 ± 2.0
207 days	6% PAC	L-malate [mM]	0.0	0.0	3.2 ± 0.5	10.6 ± 1.7	15.0 ± 0.6
		SCCs [mM]	240.6 ± 2.1	239.3 ± 1.7	219.7 ± 3.7	154.7 ± 4.4	10.8 ± 1.8
213 days	6% PAC	L-malate [mM]	0.0	0.0	0.0	0.0	0.0
		SCCs [mM]	162.2 ± 4.8	156.5 ± 3.3	170.8 ± 3.6	174.6 ± 1.8	171.8 ± 3.9

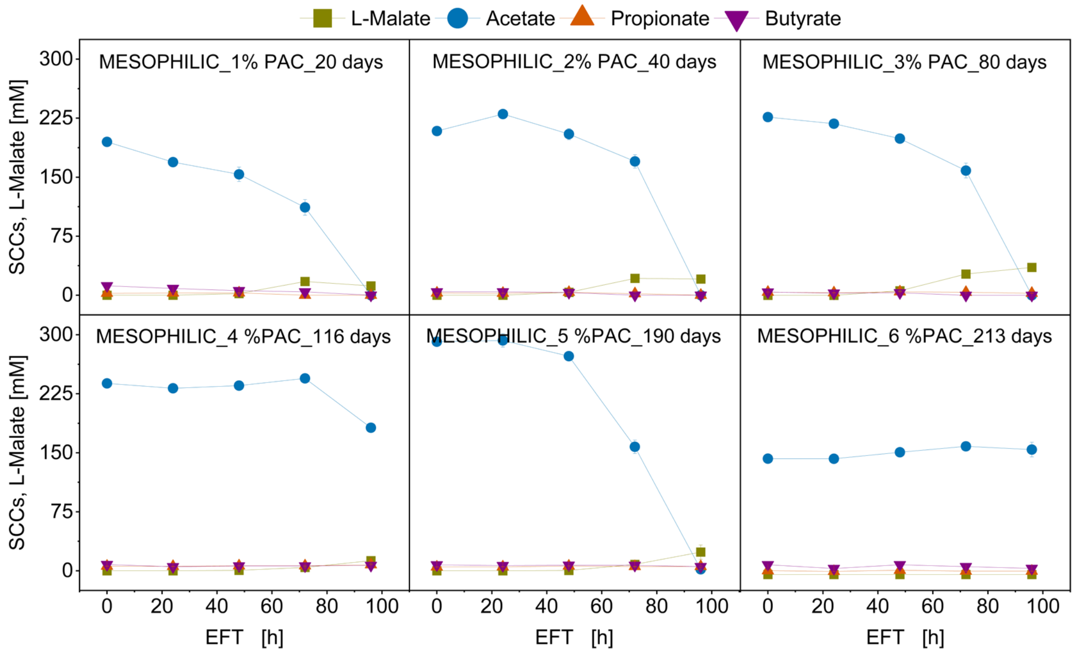

Figure S6. L-malate, acetate, propionate and butyrate concentrations over time. Numbers are mean values with standard deviations calculated from three replicates. The medium was the supernatant of the fermentation broth collected from the mesophilic reactor.

Table S5. L-malate and SCCs concentrations over time and L-malate highest yields per SCCs consumed. Numbers are mean values with standard deviations calculated from three replicates. The medium was the supernatant of the fermentation broth collected from the thermophilic reactor.

Collection time from reactor	PAC load [% v/v]	Hours from *A. oryzae* inoculation [h]	0	24	48	72	96
20 days	1% PAC	L-malate [mM]	0.0	0.0	4.2 ± 0.4	0.3 ± 0.2	0.0
		SCCs [mM]	52.3 ± 1.7	42.8 ± 3.9	22.6 ± 1.7	12.1 ± 0.7	0.0
40 days	2% PAC	L-malate [mM]	0.0	0.0	1.1 ± 0.8	4.6 ± 0.4	0.0
		SCCs [mM]	59.4 ± 0.6	57.2 ± 1.6	44.5 ± 0.7	13.7 ± 0.3	4.4 ± 1.2
80 days	3% PAC	L-malate [mM]	0.0	0.0	1.6 ± 0.6	7.4 ± 0.7	0.0
		SCCs [mM]	94.3 ± 1.6	87.5 ± 0.8	68.2 ± 2.3	12.2 ± 2.0	4.7 ± 0.6
120 days	4% PAC	L-malate [mM]	0.0	0.0	7.3 ± 0.3	13.9 ± 1.7	0.0
		SCCs [mM]	122.0 ± 1.5	105.0 ± 3.1	43.2 ± 2.8	0.0	0.0
185 days	5% PAC	L-malate [mM]	0.0	0.0	2.7 ± 0.7	2.8 ± 0.2	0.0
		SCCs [mM]	114.4 ± 5.2	104.6 ± 5.2	50.1 ± 2.0	0.0	0.0
207 days	6% PAC	L-malate [mM]	0.0	0.0	5.6 ± 0.5	4.4 ± 1.1	0.0
		SCCs [mM]	79.5 ± 1.5	66.8 ± 5.4	7.0 ± 0.1	0.0	0.0
213 days	6% PAC	L-malate [mM]	0.0	0.0	5.0 ± 0.3	3.1 ± 1.1	0.0
		SCCs [mM]	74.6 ± 0.8	66.6 ± 1.6	8.8 ± 1.2	0.0	0.0

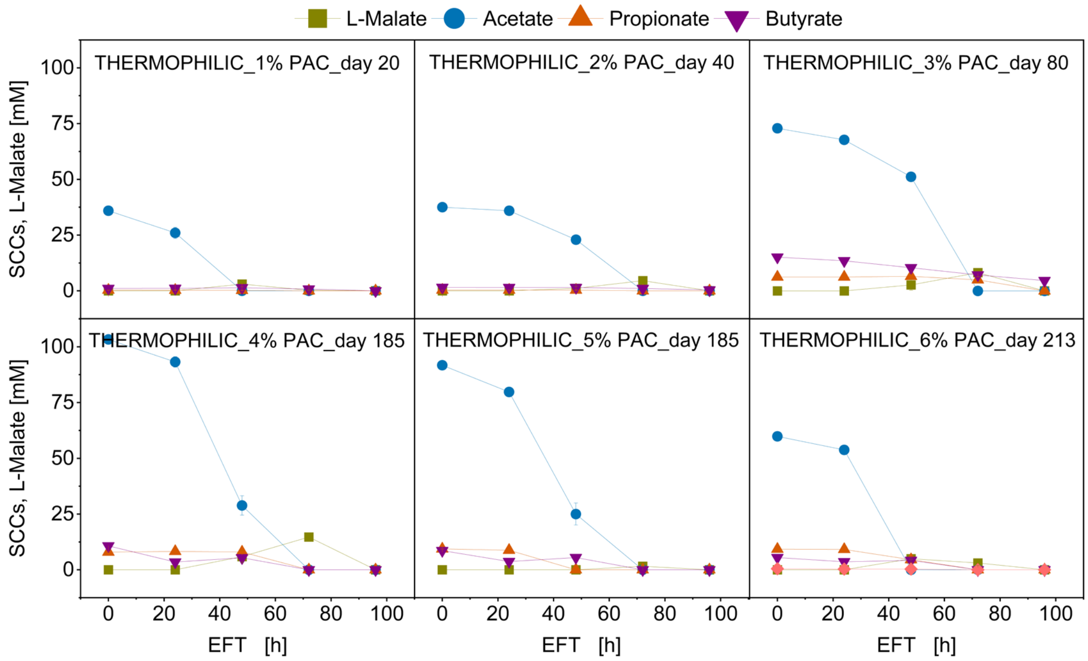

Figure S7. L-malate, acetate, propionate and butyrate concentrations over time. Numbers are mean values with standard deviations calculated from three replicates. The medium was the supernatant of the fermentation broth collected from the thermophilic reactor.

References

[1] F. Oswald *et al.*, 'Sequential mixed cultures: From syngas to malic acid', *Front. Microbiol.*, vol. 7, no. JUN, pp. 1–12, 2016, doi: 10.3389/fmicb.2016.00891.
